# The Influence of Interlocking Effects in Conjugated Polymers Synthesized by Aldol Polycondensation on Field-Effect Transistor Properties and Morphology

**DOI:** 10.1021/jacsau.5c00003

**Published:** 2025-02-27

**Authors:** Yen-Han Shih, Guan-Lin Wu, Pin-Hsiang Chueh, Jing-Chun Chen, Chu-Yen Tsai, Ting-Yu Wang, Ming-Hsuan Yu, Yi-Pei Li, Wen-Chang Chen, Chu-Chen Chueh

**Affiliations:** † Department of Chemical Engineering, National Taiwan University, Taipei 10617, Taiwan; ‡ Advanced Research Center for Green Materials Science and Technology, National Taiwan University, Taipei 10617, Taiwan

**Keywords:** organic field-effect transistor, aldol condensation, ladder-type conjugated polymers, ambipolar, DFT calculations

## Abstract

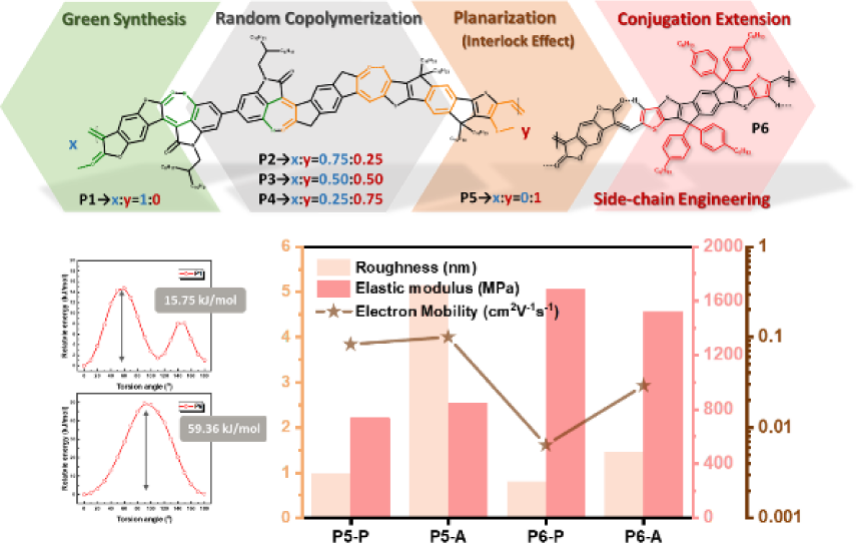

The environmental and economic drawbacks of traditional palladium-catalyzed coupling reactions in the synthesis of conjugated polymers have prompted the exploration of green alternatives. This study presents the synthesis and characterization of a series of ladder-type conjugated polymers via aldol and Knoevenagel condensation reactions, which use simple acid or base catalysts and produce only water as a byproduct. We explore the interlocking effect of the backbone and study its role in enhancing the backbone planarity, charge transport, and morphology. Intramolecular hydrogen bonding in polymers **P1** and **P5** promotes strong interlocking interactions, resulting in high electron mobilities (2.09 × 10^–2^ cm^2^ V^–1^ s^–1^ and 8.26 × 10^–2^ cm^2^ V^–1^ s^–1^, respectively) and crystalline order. In contrast, their random copolymers (**P2–P4**) exhibited disrupted interlocking effects, leading to irregular backbone distortions and reduced charge transport. **P6**, designed with a rigid ladder-type backbone and bulky side chains, exhibits an exceptional hole mobility (3.27 × 10^–1^ s cm^2^ V^–1^ s^–1^) despite an amorphous morphology, which is attributed to efficient intrachain transport. These findings demonstrate the potential of the green condensation approach in developing conjugated polymers with high charge transport properties and different morphologies through intramolecular interlocking effects.

## 1 Introduction

To date, most conjugated polymers have been synthesized via the Stille or Suzuki coupling reactions. It is well-known that the palladium (Pd) catalysts used in these coupling reactions are highly toxic and harmful to the environment. In addition, the catalyst is difficult to handle because it is susceptible to moisture and oxygen and needs to be stored in a glovebox to prevent exposure to air.

In recent years, growing concern for environmental protection has prompted scientists to explore alternative synthetic routes to reduce pollution and improve the sustainability of polymer synthesis.^1^ As a result, green synthesis methods such as aldol condensation and Knoevenagel condensation have become promising synthetic reactions for polymer synthesis, complementing the shortcomings of traditional coupling methods.^1−4^ Using simple acid or base catalysts (such as p-toluenesulfonic acid or pyridine), these reactions can be carried out in nonhalogen solvents and only produce water as a byproduct. These condensation reactions are therefore not only easy to implement, but also environmentally friendly. In addition, the bonds formed in these condensation reactions are double bonds, which enables the synthesis of ladder-type conjugated polymers.^5^ Previous studies have shown that ladder-type polymers have a high degree of planarity, which facilitates electron delocalization and regulates intramolecular and intermolecular interactions.^6,7^

Thus far, relatively few reports have been published on the synthesis of high-performance conjugated polymers via these condensation reactions compared to typical coupling reactions. Among them, BDOPV-based structures have attracted much attention due to their planar conjugated structures and superior electronic properties, making them desirable for optoelectronic applications.^8^ In this context, the synthesis of BDOPV-based analogues via condensation reactions is a promising direction.^9^ Inspired by these pioneering results, we aim to use these condensation methods to develop new conjugated polymers based on the BDOPV building blocks and expand their structures to explore derivatives with new conjugated moieties.

In this study, we first synthesized a BDOPV-based polymer (**P1**), an Indacenodithiophene (IDT)-based polymer (**P5**), and a series of their random copolymers (**P2–P4**) using aldol condensation and Knoevenagel condensation, and studied the intramolecular interactions in these polymers, as the effect of random copolymerization in such ladder-type conjugated polymers has not been studied. We then further synthesized an 3,9-bis(2-methylene-(3-(1,1-dicyanomethylene)-indanone))-5,5,11,11-tetrakis(4-hexylphenyl)-dithieno[2,3-d:2′,3′-d’]-s-indaceno[1,2-b:5,6-b’]dithiophene (ITIC)-based polymer (**P6**) and compared it with **P5** to understand the effects of the side chain on the intramolecular interactions of these polymers. Simulation results show that the oxygen atoms on the dilactone moiety can attract hydrogen atoms on the IDT or ITIC moieties, thereby forming intrachain interactions and interlocking. This interlocking effect produces good backbone planarity and promotes charge transport along the main chain, as evidenced by the high mobility of **P1** (*μ*_*e*_: 2.09 × 10^–2^ cm^2^ V^–1^ s^–1^) and **P5** (*μ*_*e*_: 8.26 × 10^–2^ cm^2^ V^–1^ s^–1^ and *μ*_*h*_: 4.26 × 10^–2^ cm^2^ V^–1^ s^–1^). However, we found that this interlocking effect is weakened by random copolymerization, resulting in irregular backbone torsions. On the other hand, by extending the core conjugation of the IDT moiety and introducing rigid side chains, we found that **P6** exhibited enhanced p-type transport behavior compared to **P5**. More interestingly, the introduction of rigid side chains greatly reduced the intermolecular interactions between polymer chains, making this rigid ladder-type polymer close to amorphous. However, the near amorphous P6 is significantly rigid due to the strong aggregation of its rigid backbone and bulky side chains. This work successfully demonstrates the advantages and prospects of using the aldol condensation or Knoevenagel condensation methods to prepare high-performance ladder-type conjugated polymers with different morphologies.

## 2 Results and Discussion

### 2.1 Synthesis and Characterization of Polymers

**Figure 1:**
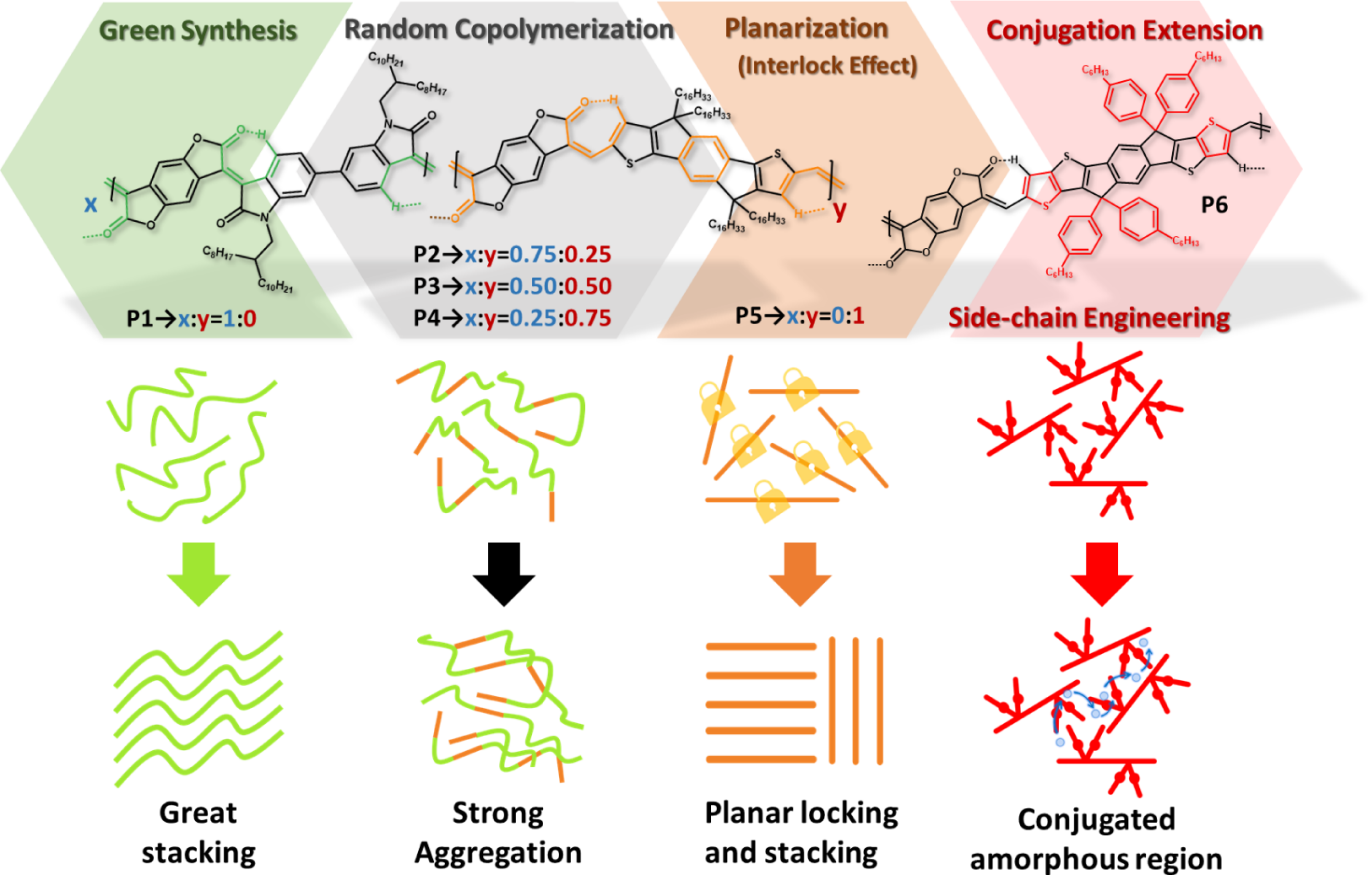
Design concepts of target polymers **P1** to **P6** and their stacking behavior as demonstrated in this study.

We used the aldol condensation reaction to synthesize conjugated polymers with enhanced environmental and sustainable properties. The detailed synthetic routes are provided in the Experimental Section. As illustrated in Figure 1, we first synthesized **P1** by polymerizing 1-(2-octyldodecyl)-6-(4,4,5,5-tetramethyl-1,3,2-dioxaborolan-2-yl)indoline-2,3-dione (**M1**) and 3,7-dihydrobenzo[1,2-b:4,5-b’]difuran-2,6-dione (**M2**). Afterward, we copolymerized **M2** with the IDT core (**M3**) to obtain **P5**, in order to increase the planarity of the target polymer. In addition, **M1**, **M2** and **M3** were randomly copolymerized in different ratios (**P2–P4**) to study the effect of random copolymerization on the charge transport properties of ladder-type conjugated polymers. Finally, **P6** was further synthesized by copolymerizing **M2** with the ITIC core to extend the conjugated backbone of the target polymer. Meanwhile, side chain was also designed to modulate the interactions and stacking characteristics between the rigid backbones, and its effect on the charge transport properties was investigated.

The^1^H NMR spectra shown in Figures S1–S10 and element analysis shown in the Supporting Information clearly indicate that our target monomers and polymers have been successfully synthesized. As summarized in Table 1, all polymers have moderate molecule weights with *M*_*n*_ values ranging from 11 to 23 kDa. We note a decreasing trend in molecular weight from **P1** to **P6**, which is apparently due to the increased rigidity of the polymer backbone, which creates steric hindrance during polymerization and reduces solubility. Normally, a lower molecular weight has a negative effect on charge transport properties, but it is exciting to see that, despite their lower molecular weights, **P5** and **P6** still have good charge transport properties.^10^Figure S11 shows the thermal gravimetric analysis (TGA) of **P1–P6**. All polymers except **P1** exhibited high thermal stability, with decomposition temperatures (*T*_*d,5%*_) above 300 °C (Table 1). Notably, *T*_*d,5%*_ increased roughly with the proportion of IDT segments from **P1** to **P5**, while **P6** had the highest *T*_*d,5%*_. The above phenomenon is related to backbone conjugation and rigidity, as rigid IDT/ITIC structures improve the thermal stability of the derived polymers by strengthening intermolecular/intramolecular interactions.^5^ We also measured the phase transitions of **P1–P6** using differential scanning calorimetry (DSC). As presented in Figure S12, there was no obvious phase transition for any of the polymers except **P5**, which may be due to its relatively low molecular weight.^11^

**Table 1:** Thermal, Optical, and Electrochemical Properties of **P1–P6**

	*M*_n_ (kDa)^a^^a^	*M*_*w*_ (kDa)^a^^a^	*Đ*a	*λ*_*max.,film*_ (nm)^b^^b^	*E*_*g*_^*opt*^ (eV)^c^^c^	HOMO (eV)^d^^d^	LUMO (eV)^e^^e^	T_d_^5%^ (°C)^f^^f^
**P1**	22.5	49.9	2.22	781	1.45	–6.02	–3.95	268
**P2**	22.4	59.4	2.65	790	1.37	–5.53	–3.76	365
**P3**	18.7	39.0	2.08	733	1.37	–5.53	–3.79	318
**P4**	12.1	25.5	2.11	733	1.54	–5.54	–3.56	366
**P5**	11.4	22.5	1.96	740/667	1.59	–5.55	–3.61	376
**P6**	12.8	24.3	1.90	710/647	1.64	–5.45	–3.72	390

a *M*_*n*_ and *M*_*w*_ values were measured by GPC with *o*-dichlorobenzene as the eluent.

b Maximum of the film’s UV–Vis absorption.

c Optical *E*_*g*_ value was evaluated based on the absorption onset of the polymer film deposited on a quartz substrate.

d CV was recorded using Fc/Fc^+^ as the internal potential reference.

e Estimated by LUMO = HOMO + *E*_*g*_.

f Determined from the TGA curve at 5% weight loss.

### 2.2 Optical and Electrochemical Properties

**Figure 2:**
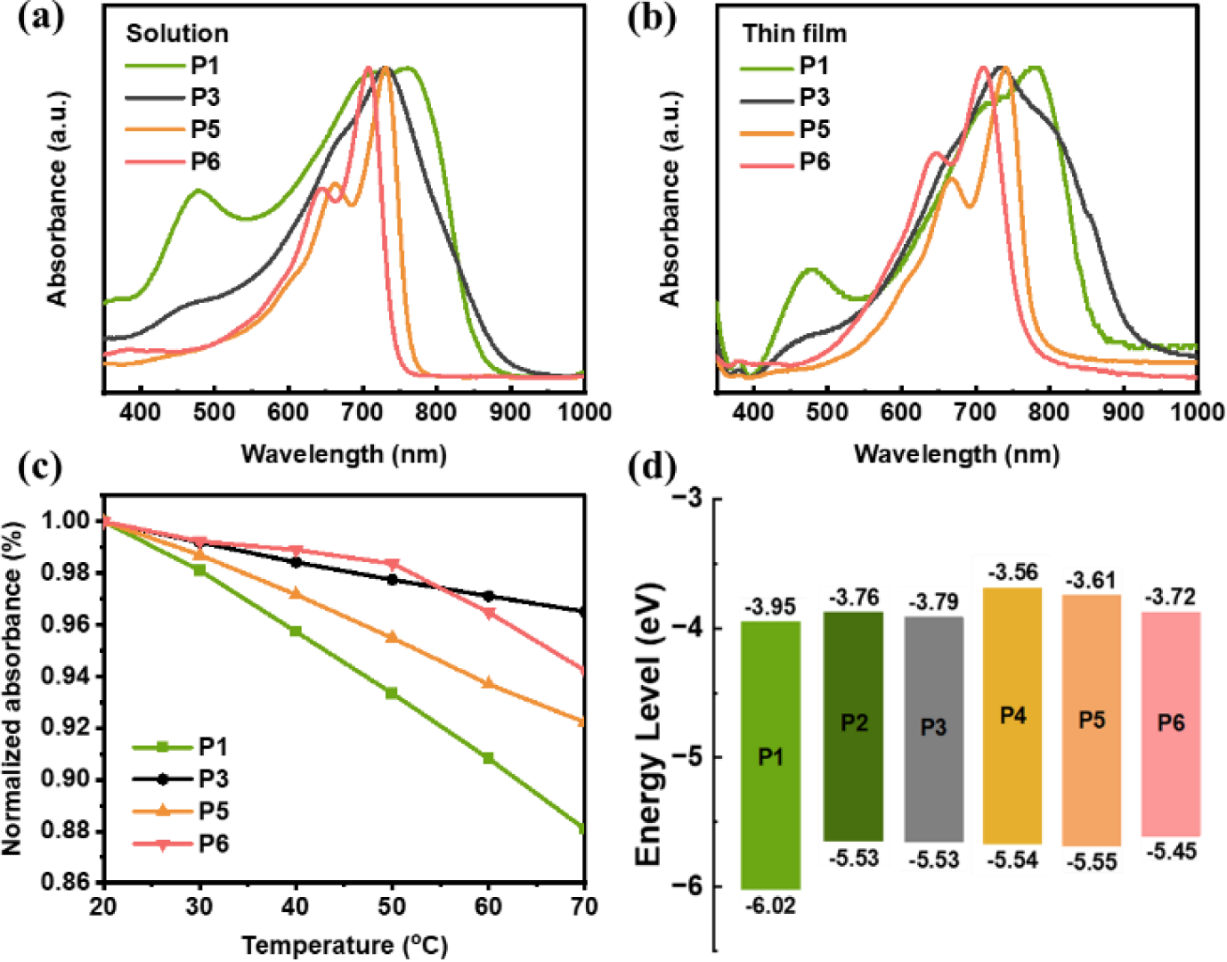
Normalized UV–vis absorption spectra of (a) the solutions and (b) films of the studied polymers. (c) Temperature-dependent absorption spectra of the polymer solutions with normalized absorbance. (d) Energy-level diagram of **P1–P6**.

We measured the UV–vis absorption spectra of these polymers in solution and film states to study their optical properties of these polymers, as shown in Figure 2a,b, respectively. For the sake of simplicity, the results of **P2** and **P4** are not included in the discussion. However, their corresponding data is provided in Figure S13. As shown, **P1** displays the typical UV–vis spectrum of a conjugated copolymer. The absorption band between 400 and 600 nm is attributed to π–π* transitions, while the absorption band between 600 and 900 nm results from intramolecular charge transfer (ICT) between electron-donating and electron-withdrawing monomer units. The ICT of the polymer is enhanced in the solid state due to the intense aggregation, leading to a red shift and narrower bandgap in the film UV–vis spectra.^12^ Notably, **P3** exhibits a broad shoulder from 730 to 900 nm (Figure 2b), indicating a more irregular aggregation behavior of **P3** compared to its parent polymers **P1** and **P5**. This result demonstrates that the random copolymerization has a significant influence on such ladder-type conjugated polymers.

We then measured the temperature-dependent UV absorption spectra of the **P1–P6** solutions to study the deaggregation behavior of these polymers. To compare the aggregation behavior between the different polymers, we calculated the relative absorbance of the highest peak for each polymer and plotted the results in Figure 2c. Again, we have not shown the results of **P2** and **P4** in order to simplify the comparison. Due to the deaggregation of the polymers, the absorbance of the polymers decreases with increasing temperature and undergoes a slight blue shift.^2^ Clearly, the relative absorbance of these four polymers decreases to different degrees with temperature. When the temperature is below 50 °C, the relative absorbance of **P6** decreases less than that of all other polymers, but when the temperature rises further, its relative absorbance suddenly decreases. This is because the structure of **P6** is stiffer and bulkier than that of other polymers, and its rigid side chains cause it to form a strongly entangled structure, which is more prone to aggregate at lower temperatures. When the temperature rises to 50 °C, **P6** gains enough energy to deaggregate, so it quickly becomes loose, causing the relative absorbance to drop rapidly. In addition, **P3** has the weakest deaggregation behavior, indicating that its irregular structure has a strong aggregation behavior. On the other hand, **P1** exhibits the most obvious deaggregation behavior due to the lower rigidity of its backbone.

The bandgap (*E*_*g,opt*_) values of **P1–P6**, evaluated from the absorption onset in the film UV–vis spectra, were 1.45, 1.37, 1.37, 1.54, 1.59, and 1.64 eV, respectively. The highest occupied molecular orbital (HOMO) and lowest unoccupied molecular orbital (LUMO) energy levels of each polymer, as measured by cyclic voltammetry (CV) (Figure S14), are summarized in Figure 2d. The corresponding bandgaps (*E*_*g,CV*_) values for **P1–P6**, calculated using the equation of LUMO = *E*_*g,CV*_ + HOMO, were 2.07, 1.77, 1.74, 1.98, 1.94, and 1.73 eV. There is no clear trend in the energy levels of the random copolymers (**P2–P4**). We note that **P1** should be an n-type polymer because it has good reduction ability but poor oxidation ability (Figure S14).^13^ In addition, the planar and rigid backbones will reduce the energy gap, which explains why the bandgaps of **P5** and **P6** are lower than that of **P1**.

### 2.3 Molecular Simulation

**Figure 3:**
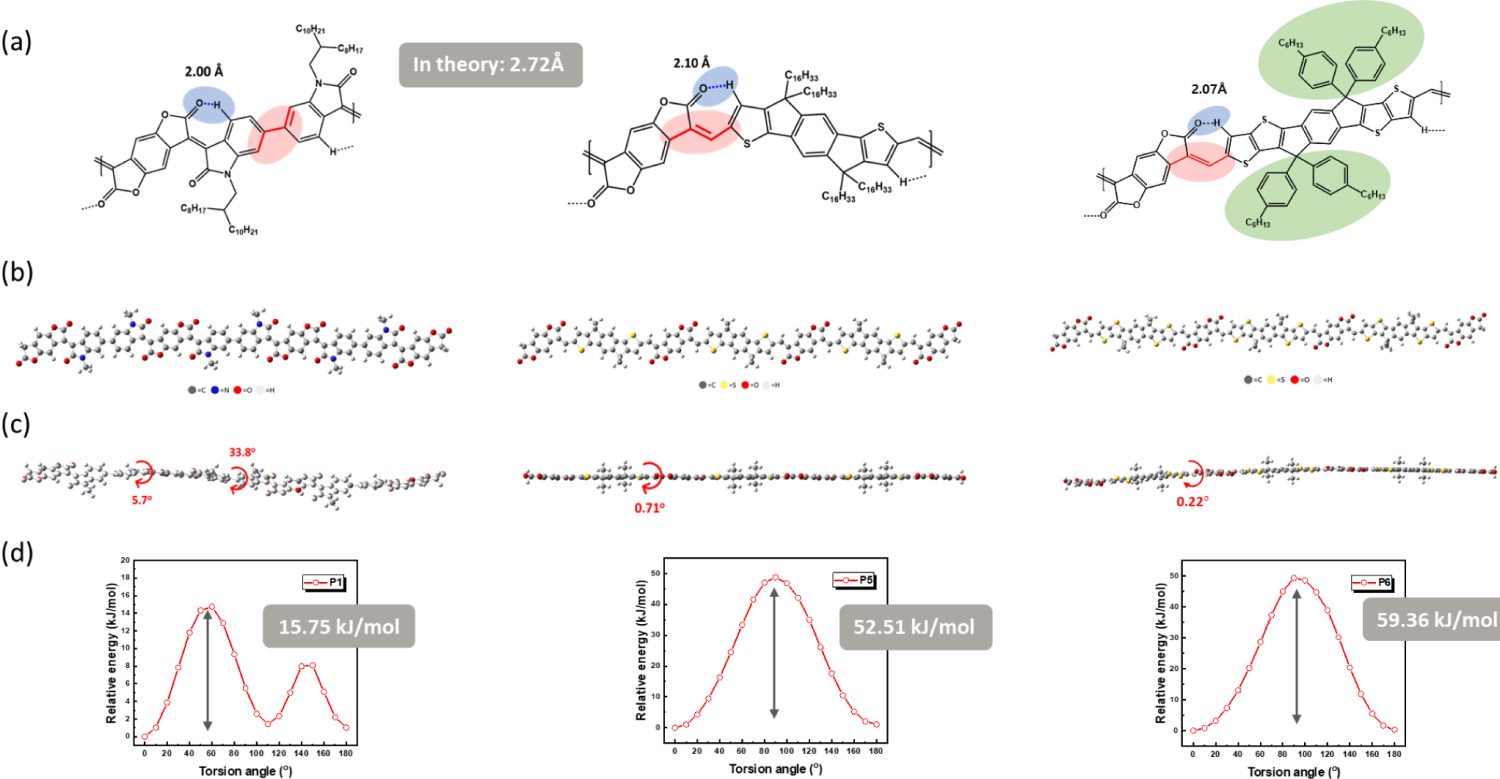
Molecular simulations of **P1**, **P5** and **P6**. (a) Chemical structures and atomic distances. (b) Top view, (c) side views, and (d) labeled dihedral angle energy scans of **P1**, **P5** and **P6**.

To elucidate the configurations and conformations of **P1**, **P5** and **P6**, we performed density functional theory (DFT) calculations on three repeating units of each polymer, where the alkyl chains were replaced by methyl chains to simplify the calculations. The Gaussian 09 program and the B3LYP/6-31G(d) basis set were used. Figure 3a–c presents the front and side views of **P1, P5** and **P6**. Due to the rotational freedom of the single bond in **M1**, the maxima dihedral angle in **P1** is 33.8°. Interestingly, the interatomic distance between the oxygen atom on the lactone group in **M2** and the hydrogen atom in **M1** is 2.00 Å, which is shorter than the sum of their theoretical van der Waals radii (2.72 Å). This indicates the formation of a noncovalent O–H intrachain bond. Therefore, the dihedral angle between **M1** and **M2** is only 5.7°, and similar results are also found for **P5** and **P6**. **P5** and **P6** are highly planar conjugated polymers, and the dihedral angles between the two monomers reduced to 0.71° and 0.22°, respectively.

To further investigate the planarity and intramolecular interactions of the polymers, we scanned the total energy of the dihedral angle rotation starting from the lowest energy point. As portrayed in Figure 3d, the total energy of **P1** remains lowest at 0° and 110°, with an energy barrier of 15.75 kJ/mol. This means that **P1** is susceptible to thermal perturbation and prone to twisting on the backbone. In contrast, the lowest energy of **P5** and **P6** is maintained at 0° and 180°, respectively, and the energy barriers between states are 52.51 and 59.36 kJ/mol, respectively. The results show that **P5** and **P6** are unlikely to change their conformations, and even if they do, the entire backbone will remain flat and rigid.

Given the high rigidity of **P5** and **P6**, we further investigated their reorganization energies. The reorganization energy is the energy required to distort a neutral molecule into a charge-transfer state, and it may to be the kinetic limit step for ICT.^14,15^ In principle, reducing the reorganization energy can reduce energy disorder and improve ICT efficiency.^16^ Here, the reorganization energy is calculated by estimating the commensurate energy of positively or negatively charged oligomers. The detailed calculation method is described in the Supporting Information, and the relevant calculation results are summarized in Tables S1 and S2. The estimated λ_hole_ values for **P5** and **P6** are 115.2 and 94.3 meV, respectively, and their λ_electron_ values are estimated to be 94.3 and 102.4 meV. It is worth noting that the λ_electron_ value of **P5** is lower than that of **P6**, indicating that **P5** has stronger electron transport properties. The λ_electron_ value of **P6** also exceeds its λ_hole_ value, indicating that it has stronger p-type transport characteristics.

### 2.4 Morphological Characterization

**Figure 4:**
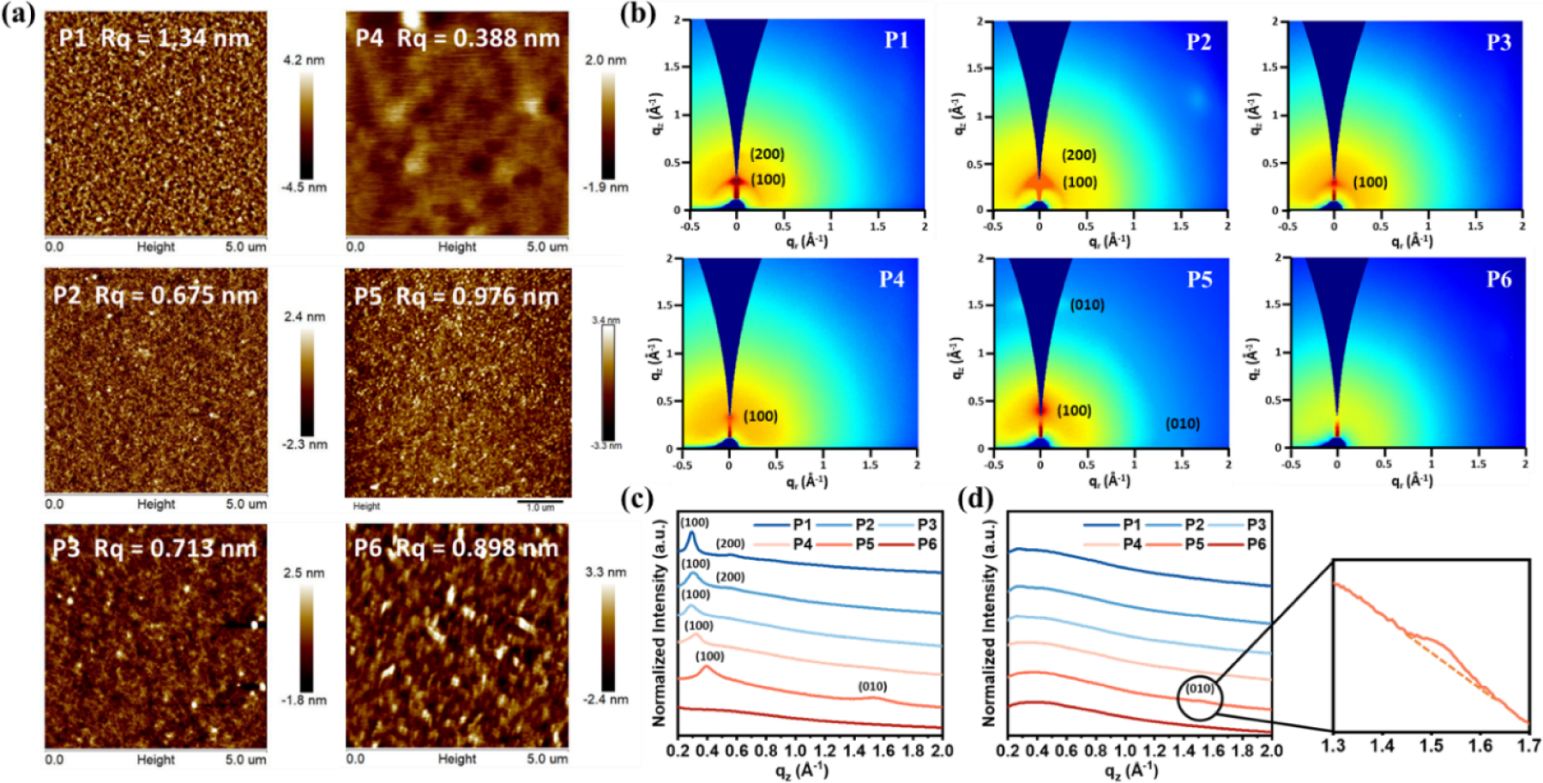
(a) AFM height images and the corresponding surface roughness of the **P1–P6** films. (b) 2D GIWAXS patterns of the **P1–P6** films, and 1D X-ray scanning profiles extracted in (c) the OOP and (d) IP directions.

The film properties of these polymers were then analyzed to gain insight into solid-state aggregation and crystalline structure. We first analyzed the surface topologies of the **P1–P6** films using atomic force microscopy (AFM), as presented in Figures 4a and S15. As shown in Figure 4a, the surface roughness (*R*_*q*_) of most polymer films was low and flat, indicating uniform morphology. However, it is worth noting that **P6** has the most white spots on its surface, confirming that **P6** has stronger aggregation behavior due to its rigid backbone and bulky side chain. In addition, the *R*_*q*_ values of the **P1** and **P5** films are much higher than those of the other films, indicating that they form a higher crystalline structure,^17^ which will be discussed further below.

To investigate the crystalline structure of these films in more detail, we performed grazing-incidence wide-angle X-ray scattering (GIWAXS) analysis. Figure 4b shows the two-dimensional (2D) patterns of the **P1–P6** films, and the extracted one-dimensional (1D) results in the out-of-plane (OOP) and in-plane (IP) directions are presented in Figure 4c,d, respectively. The main features of the crystallographic properties, such as *d*-spacing, π–π stacking distance, and crystallographic coherence length (CCL), are calculated and summarized in Table S3. As can be seen in Figure 4b, although the backbone of **P1** has a 33° dihedral angle, it is still able to form a good crystalline structure, with obvious (100) and (200) peaks in the OOP direction and *d*-spacing of 21.44 Å. This result shows that ordered interchain stacking behavior can still be formed in a well-organized twisted system. In contrast, **P5**, which has a highly coplanar structure, exhibits a (100) peak in the OOP direction and a (010) peak in the OOP and IP directions. The (100) peak indicates the presence of a lamellar stacking structure, while the (010) peak suggests the presence of a π–π stacking structure. These results show that a bimodal system is formed due to the coexistence of face-on and edge-on structures, enabling charge carriers to transport in both the vertical and horizontal directions. As a result, three-dimensional (3D) charge transport paths are formed, which improves charge transport performance.^18^ It is also worth noting that the *d*-spacing of **P5** (15.81 Å) is significantly lower than that of **P1**, exhibiting a more compact lamellar stacking behavior, which indicates that the coplanarity of the backbone has an impact on the packing density of the crystalline system.

Although **P1** and **P5** both exhibit good structural stacking behavior, their random copolymers (**P2–P4**) have significantly lower crystallinity. As can be seen in Figure 4b and Table S3, **P2–P4** all exhibited weak lamellar stacking behavior and large *d*-spacings (20.59 Å, 21.42 Å, 19.81 Å). This relatively disordered structure is due to irregularly distorted backbones interfering with the formation of close packing. The irregular structure therefore leads to disordered aggregation behavior, increasing the entanglement between the polymer chains and forming more amorphous region, as illustrated in Figure 1.

Interestingly, **P6** has an extended and planar conjugated backbone, yet its morphology is almost amorphous. This can be attributed to its bulky side chains, which lead to predominantly disordered short-range aggregates rather than long-range ordered stacking structure, despite the strong intermolecular interactions of the planarized backbone.^2,19^ This observation is in good agreement with the temperature-dependent UV–vis spectroscopy (Figure 2c), which describes the easy aggregation behavior of **P6**. The above results clearly demonstrate the important influence of side-chain design. The neat side-chain arrangement facilitated by the planarized backbone observed in **P5** is disrupted in **P6** due to the steric hindrance of the rigid and bulky side chains, resulting in a nearly amorphous character.

### 2.5 Field-Effect Transistor Performance

**Figure 5:**
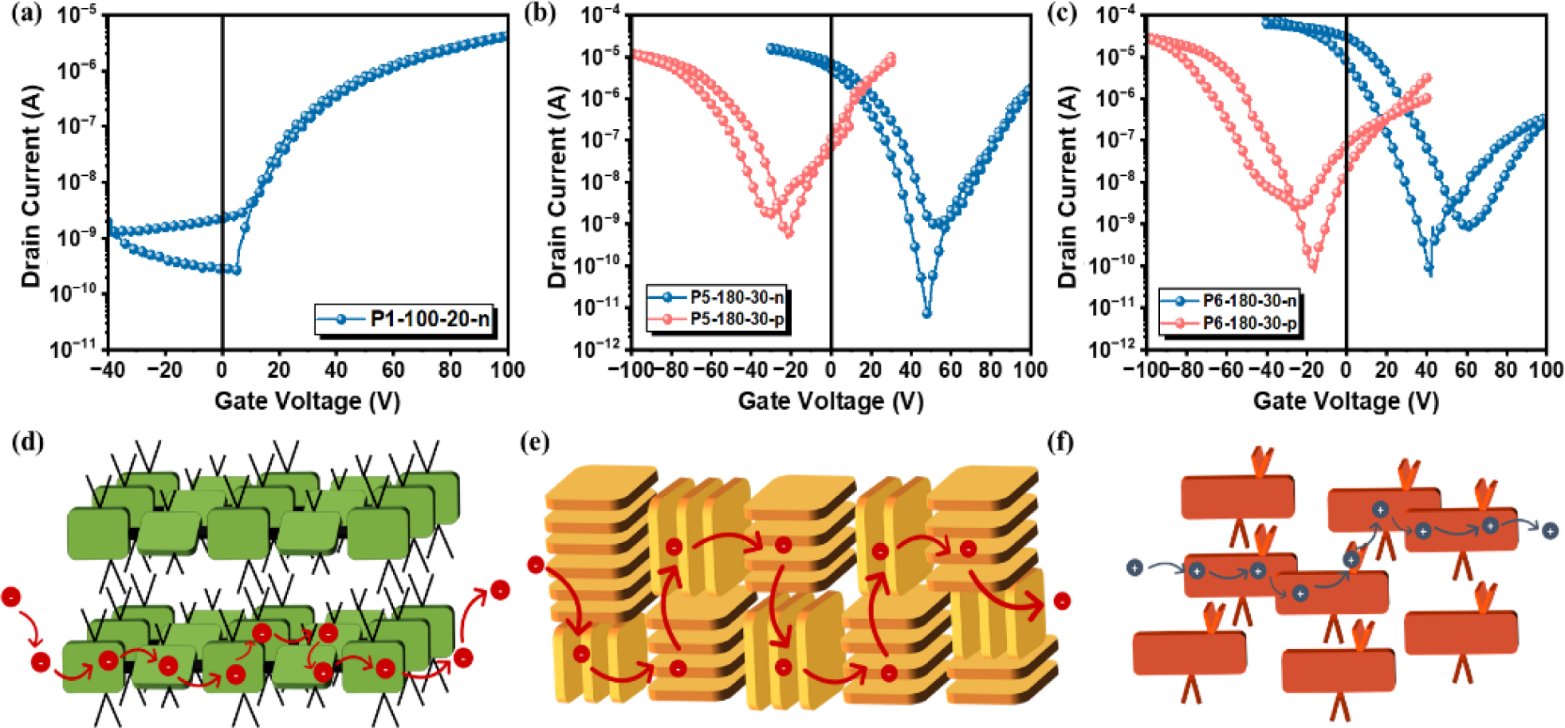
Transfer characteristics and corresponding charge transport mechanisms of the devices based on (a, d) **P1**, (b, e) **P5**, and (c, f) **P6**.

We finally investigated the charge transport characteristics of **P1–P6** in OFETs with bottom gate (BG)/top contact (TC) structures, and the details of device fabrication are described in Supporting Information. Figures 5, S15, and S16 display the transfer and output characteristics of **P1–P6**, and the relevant device performances are summarized in Table 2. As shown, **P1** is the only polymer that exhibits unipolar n-type electronic transport property, with electron mobility of 2.09 × 10^–2^ cm^2^ V^–1^ s^–1^. This high mobility is attributed to its order crystalline structure, which facilitates interchain charge transfer (Figure 5d). In addition, the low-lying HOMO level of **P1** forms a large energy barrier for hole injection (Figure 2d), which contributes to its unipolarity.

**Table 2:** FET Characteristics of **P1–P6** Devices After Annealing^a^

Material	T (°C)	T (min)	μ_e_ (cm^2^ V^–1^ s^–1^)	*I*_on_/*I*_off_	*V*_th_ (V)	μ_h_ (cm^2^ V^–1^ s^–1^)	*I*_on_/*I*_**off**_	*V*_th_ (V)	μ_e_/μ_h_
**P1**	100	20	2.09 × 10^–2^	3.4 × 10^–4^	13.2	×	×	×	x
**P2**	180	30	2.17 × 10^–4^	10^3^ ∼ 10^5^	17.2	5.04 × 10^–4^	10^2^∼10^3^	–68.7	4.31
**P3**	150	30	2.75 × 10^–5^	10^2^ ∼ 10^3^	34.1	2.18 × 10^–4^	10^2^∼10^3^	–42.9	0.13
**P4**	180	30	2.95 × 10^–5^	10^4^ ∼ 10^6^	40.6	1.60 × 10^–2^	10^4^∼10^5^	–42.7	1.84 × 10^–3^
**P5**	180	30	8.26 × 10^–2^	10^3^ ∼ 10^4^	70.0	4.26 × 10^–2^	10^3^∼10^4^	–40.6	1.94
**P6**	180	30	6.44 × 10^–3^	10^5^ ∼ 10^7^	74.9	3.27 × 10^–1^	10^5^∼10^7^	–48.8	1.97 × 10^–2^

a The mobility values are averaged from at least 12 devices made from 3 different batches.

Except for **P1**, other polymers (**P2–P6**) all exhibit ambipolar charge transport behavior due to their appropriate energy levels. As shown in Figure 2d, the HOMO energy levels of **P2–P6** are relatively high, at ∼−5.5 eV, ensuring that these polymers will not encounter a large energy barrier during hole injection. Meanwhile, the LUMO energy levels are close to or below −3.6 eV, ensuring the potential for electron injection. However, as can be seen from Table 2, the charge transport properties of the random polymers **P2–P4** are relatively poor, with electron and hole mobilities (*μ*_*e*_, *μ*_*h*_) of (2.17 × 10^–4^, 5.04 × 10^–5^), (2.75 × 10^–5^, 2.18 × 10^–4^), and (2.95 × 10^–5^, 1.60 × 10^–2^) cm^2^ V^–1^ s^–1^, respectively. The low mobility of these random copolymers is attributed to their irregularly twisted backbones. The irregularly twisted backbones lead to disrupted conjugation and disordered entangled structures, which in turn negatively affect the intramolecular and intermolecular charge transport. As a result, **P2–P4** have relatively poor properties. Encouragingly, **P5** exhibits impressive performance in both electron and hole transport, with average mobilities (*μ*_*e*_, *μ*_*h*_) of (8.26 × 10^–2^, 4.26 × 10^–2^) cm^2^ V^–1^ s^–1^, respectively. The bimodal crystalline structure shown in Figure 5e has 3D charge transport characteristics, providing more flow pathways for charge carriers, thereby achieving high mobilities.^20^ This result proves that the carrier mobility of the conjugated polymer can be further improved by enhancing the structural planarity, resulting in compact stacking behavior and appropriate intramolecular interactions. Last but not least, although **P6** has an amorphous character, its carrier mobility is still very impressive, with mobilities (*μ*_*e*_, *μ*_*h*_) of (6.44 × 10^–3^, 3.27 × 10^–1^) cm^2^ V^–1^ s^–1^, respectively. The results show that efficient intrachain transport, which relies on intensive backbone aggregation and a ladder-type backbone, successfully compensates for the loss of interchain charge transport, thereby ensuring excellent charge transport properties (Figure 5f).^2^ In fact, previous studies have shown that if the molecular weight of a polymer is high enough, the polymer will bridge short-range aggregates and act as a tie-molecule.^21,22^ Therefore, although **P6** lacks a long-range ordered structure, its efficient intramolecular charge transport behavior, combined with the linking tie-chains between aggregates, can still ensure its transport capability.

The difference in charge transport properties between **P5** and **P6** is very significant, with **P5** exhibiting n-type preferential transport characteristics and **P6** showing p-type preferential characteristics. This difference obviously stems from the difference in electron and hole reorganization energies in the two polymers (as discussed earlier).^2,23^Figure S18 shows the relationship between the reorganization energy and mobility of **P5** and **P6**. Specifically, **P5** has a lower λ_electron_ value and **P6** has a lower λ_hole_ value. Therefore, electrons encounter less resistance when moving in **P5**, while the transport of holes in **P6** is more favorable. The *μ*_*e*_/*μ*_*h*_ ratios for **P5** and **P6** are 1.94 and 1.97 × 10^–2^, respectively, which clearly shows the effect of the reorganization energy on the charge transport behavior.

### 2.6 Solvent Additives Effect

Compared with previous studies on conjugated polymers synthesized by the aldol condensation method,^1−4,7,24−30^ the charge transport properties of **P1**, **P5**, and **P6** are quite good, as summarized in Figure S19. In view of this result, we expect to further improve the properties of **P5** and **P6** by using solvent additives to optimize their crystalline structures and aggregation behavior. By adding a high-boiling point (b.p.) solvent to the system, the crystallization rate can be slowed down, giving the polymer more time to form a better arrangement, especially for rigid polymers. Here, we chose 3% 1-chloronaphthalene (CN) and 10% 1,2-dichlorobenzene (ODCB) as the solvent additives for **P5** and **P6**, respectively. As shown in Table 3, the electron *μ*_*e*_ values of both polymers increased slightly after the addition of the solvent additives, while the *μ*_*h*_ values remained similar. Figure S20 shows that the energy levels of the polymers are similar after the addition of the solvent additive, so the observed enhancement effect is not caused by a change in energy levels.

**Table 3:** Changes in the FET Characteristics of **P5** and **P6** After the Addition of Solvent Additives^a^^b^

	**P5**		**P6**	
Condition	Pristine	3%CN	Pristine	10%ODCB
**μ**_**e**_ (**cm^2^ V^–1^ s^–1^**)	0.083 ± 0.028 (0.15)	0.10 ± 0.023 (0.16)	0.0064 ± 0.0029 (0.014)	0.029 ± 0.011 (0.051)
**μ**_**h**_ (**cm^2^ V^–1^ s^–1^**)	0.043 ± 0.013 (0.069)	0.031 ± 0.011 (0.049)	0.33 ± 0.043 (0.43)	0.20 ± 0.098 (0.46)

a Mobility values were averaged from at least 12 devices made from 3 different batches.

b The values in brackets are the maximum mobility values.

**Figure 6:**
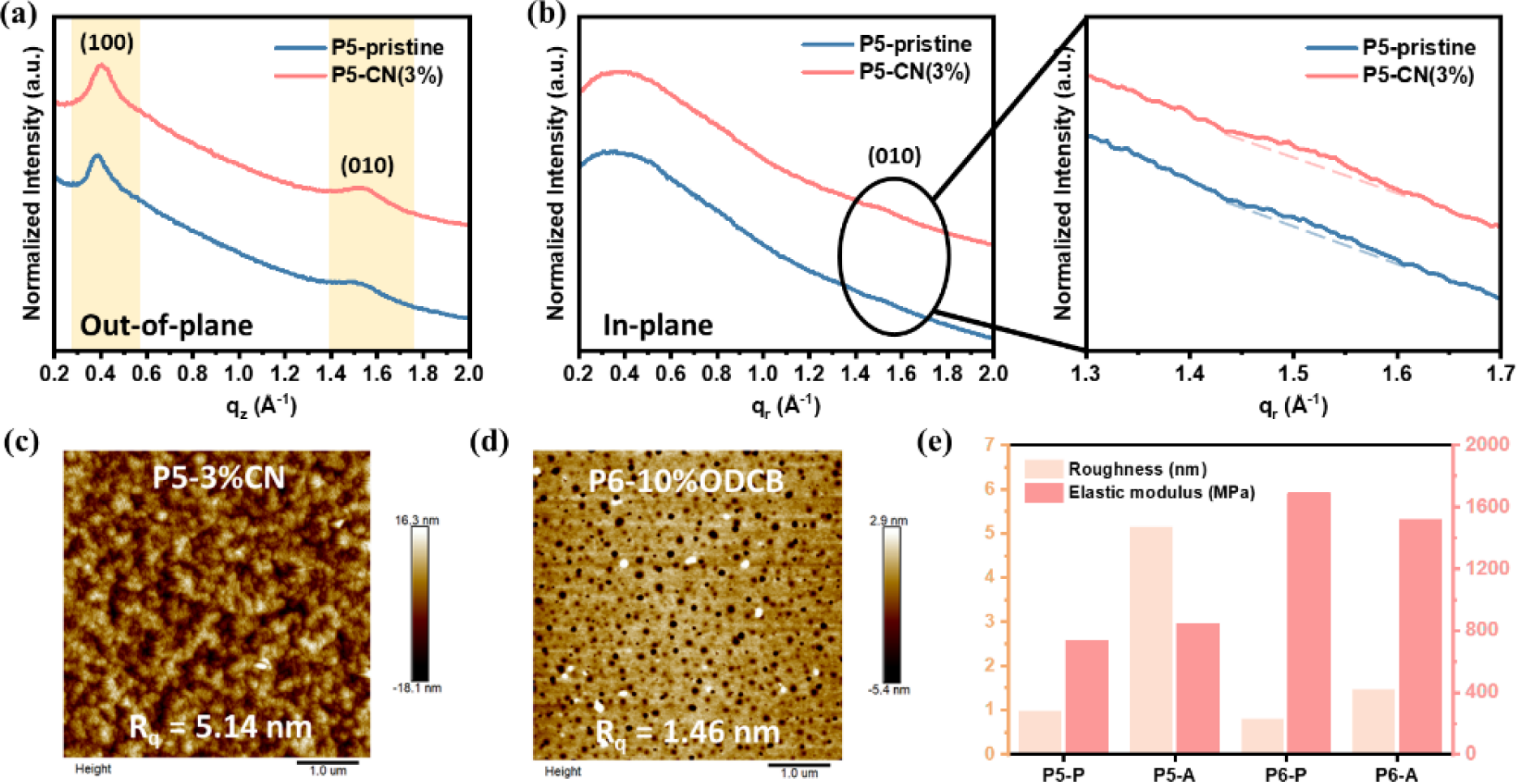
(a) GIWAXS-derived OOP and (b) IP 1D-scanning curves of **P5** in the pristine state and after the addition of the solvent additive. AFM height images of (c) **P5** and (d) **P6** with solvent additives and the corresponding surface roughness. (e) Changes in the roughness and elastic modulus of **P5** and **P6** after the addition of solvent additives.

To understand the reasons for the improved performance, GIWAXS, AFM, and Derjaguin-Muller-Toporov (DMT) model analyses were carried out to understand the effects of the solvent additives on crystallinity, surface morphology, and surface rigidity. Figure 6a,b and Table S4 show that the crystallinity of **P5** increased, and the lamellar and the π–π stacking distances decreased, indicating a more compact stacking behavior. An increase in crystallinity is also observed in Figure 6c, where the AFM profile shows greater roughness and a distinct nanocrystalline morphology, indicating that the addition of the solvent additive successfully promoted chain alignment. This higher crystalline structure ultimately results in a harder surface, giving **P5** a higher elastic modulus value (Figure 6e), which is related to the mechanical properties discussed in the next section.

After the addition of the solvent additive, the GIWAXS profile of **P6** still shows a nearly amorphous morphology (Figure S21). This result show that there is no long-range ordered structure the polymer film, indicating that it is still a system dominated by aggregates even after the addition of the additive. It is worth noting that, as shown in Figure 6e, the elastic modulus of the **P6** film decreases with the addition of the solvent additive. This suggests that some changes have occurred in the strongly entangled structure of the rigid backbone and side chains, thereby improving the charge transport behavior.

### 2.7 Mechanical Property Characterization

**Figure 7:**
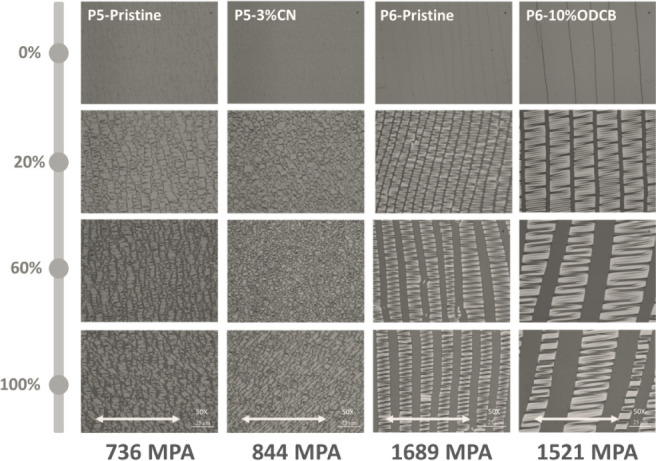
OM images of the transferred and stretched polymer films of **P5** and **P6** in their original state and with solvent additives, with strain levels from 0% to 100%. The scale bar is 25 μm. The elastic modulus obtained from DMT model is listed below the image.

It is generally believed that the amorphous regions in the semicrystalline structure can promote energy dissipation during stretching, and several studies have also shown that a near-amorphous form is beneficial for improving stretchability.^31,32^ Given the different crystallinities of **P5** and **P6**, we therefore proceeded to study and compare their mechanical properties in order to explore their potential for stretching applications. To do this, the transferred and stretched polymer films were prepared and the surface morphology after stretching was examined under an optical microscope (OM), as shown in Figure 7.

According to DMT analysis, the elastic moduli of **P5** in its original state and after the addition of a solvent additive were 736 and 844 MPa, respectively. Such a high elastic modulus indicates that **P5** is highly rigid, due to its planarized backbone and highly crystalline structure. Therefore, the film will crack at low strain. Compared with the original film, the film with a solvent additive has a higher crack density, which is related to the increase in crystallinity. On the other hand, despite its amorphous character, the elastic modulus of **P6** is unexpectedly very high, with 1689 MPa for the original film and 1521 MPa after the addition of the solvent additive. This high elastic modulus indicates that the **P6** film is significantly rigid, which is caused by the strong aggregation of its rigid backbone and bulky side chains. The surface of the polymer film is therefore not a soft, amorphous surface, but a brittle surface consisting of tightly entangled polymer chains. This tightly entangled, rigid structure reduces the ability of the polymer to rotate and rearrange—a key factor in dissipating tensile stress. As a result, cracks can easily form even at low strain levels. In addition, when a solvent additive was introduced, cracks can propagate more quickly and grow larger, spreading along the direction of the surface pores (Figure 7). This result shows that the amorphous character is not absolutely correlated with high ductility. When the structure of the material is too rigid, the resulting film becomes fragile and cannot effectively withstand the applied stresses.

## 3 Conclusion

This study demonstrates the great potential of the aldol and Knoevenagel condensation reactions as an environmentally sustainable alternative for the synthesis of high-performance ladder-type conjugated polymers. Through the systematic synthesis and characterization of a series of polymers (**P1–P6**), we revealed the importance of backbone planarity and intramolecular interlocking in enhancing charge transport and morphological stability. **P1** and **P5** have good crystalline structures with interlocking effects through intramolecular hydrogen bonding, thus exhibiting high electron mobilities of 2.09 × 10^–2^ cm^2^ V^–1^ s^–1^ and 8.26 × 10^–2^ cm^2^ V^–1^ s^–1^, respectively. In contrast, their random copolymers (**P2–P4**) exhibited inferior charge transport behavior due to the irregular backbone twist that disrupts the interlocking effect. Interestingly, **P6**, with its rigid backbone and bulky side chains, achieved an excellent hole mobility (3.27 × 10^–1^ cm^2^ V^–1^ s^–1^) due to efficient intrachain transport, despite being amorphous in morphology. Morphological analysis confirmed that increasing the backbone rigidity enhanced the crystalline alignment in **P5** but led to amorphous aggregation in **P6**. Furthermore, solvent additives improved the packing density of **P5** and enhanced charge transport, while reducing the entanglement of the rigid chains in **P6**, despite the limited understanding of its morphology. Mechanical measurements indicated that high rigidity and hardness, while beneficial for electronic properties, may reduce ductility, as demonstrated by the brittle behavior of both **P5** and **P6** films under strain. This work demonstrates the potential of the green condensation approach in the development of conjugated polymers with high charge transport properties in different morphologies. Based on these findings, future efforts may focus on modifying the alkyl chains in **P6** while retaining the strong hydrogen-bonding groups that promote the interlocking effect in the backbone, in an attempt to strengthen the interchain stacking and further improve its charge transport efficiency.

## 4 Experimental Section

### 4.1 Materials

All chemicals were purchased from Alfa Aesar, Sigma-Aldrich, Tokyo Chemical Industry, Acros Organics, and Lumtech Inc., and were used as received without further purification. Detailed synthetic routes for monomers including 6-bromo-1-(2-octyldodecyl)indoline-2,3-dione (**1**), 1-(2-octyldodecyl)-6-(4,4,5,5-tetramethyl-1,3,2-dioxaborolan-2-yl)indoline-2,3-dione (**2**), 1-(2-octyldodecyl)-6-(4,4,5,5-tetramethyl-1,3,2-dioxaborolan-2-yl)indoline-2,3-dione (**M1**), and 3,7-dihydrobenzo[1,2-b:4,5-b]difuran-2,6-dione (**M2**) are described in Supporting Information and are based on methods reported in the literature.

### 4.2 Device Fabrication

We fabricated bottom-gate/top-contact (BG/TC) FETs for studied polymers. Highly n-doped silicon (100) wafers were selected as substrates, with gate dielectric layers of 300 and 100 nm thick SiO_2_ and capacitances of ∼10 nF/cm^2^ and 29 nF/cm^2^, respectively. An octadecyltrichlorosilane (ODTS) self-assembled monolayer was coated on the SiO_2_ surface to improve the surface roughness and promote the formation of the crystal structure of studied polymers. The active layer was prepared by spin-coating a polymer solution (5 mg/mL in chloroform) at 2000 rpm for 60 s, followed by annealing at an optimized temperature for 30 min, with the annealing temperature varying depending on the polymer. Finally, a 60 nm thick Au was deposited through a shadow mask to define the top contact source/drain electrodes, with a channel length (*L*) of 100 μm and a channel width (*W*) of 2000 μm. For the case using solvent additives, the spin-coating process is slightly different due to their high boiling point. In the 3% CN-P5 system, the polymer film was prepared at 2000 rpm for 120 s, while in the 10% ODCB-P6 system, the polymer solution was placed on the substrate for 10 s, and then spin-coated at 1000 rpm for 90 s. The characteristics of FETs were measured in a N_2_-filled glovebox using a Keithley 4200-SCS semiconductor parameter analyzer (Keithley Instruments Inc.). The mobility value is calculated from the transfer curve at the saturated regime according to the following equation:
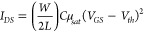
where *I*_*DS*_ is the drain current, *W* and *L* are the channel width and channel length, respectively, *C* is the areal capacitance of the dielectric layer, *μ*_*sat*_ is the mobility in the saturation region, and *V*_*GS*_ and *V*_*th*_ are the gate voltage and threshold voltage, respectively. The *μ*_*sat*_ value of the device is then determined from the correlation between the square root values of *I*_*d*_ and *V*_*GS*_.

### 4.3 Characterization

Microwave polymerization was carried out using a Biotage microwave reactor under a nitrogen atmosphere with a 5 mL vessel sealed. The number-average molecular weight (*M*_*n*_) of polymers was measured using a gel permeation chromatograph (GPC) with Enshine SUPER CO-150, and polystyrene gel columns (Stryagel HR 2 and styragel 4) eluted with o-dichlorobenzene at a flow rate of 1.0 mL/min and calibrated with standard polystyrene. Thermogravimetric analysis (TGA) and differential scanning calorimetry (DSC) were performed using TA Instruments TGA55 and DSC25 at a heating rate of 10 °C/min. UV–vis-NIR absorption spectra were recorded using a Hitachi U-4100 spectrophotometer. Temperature-dependent absorption spectra were measured between 20 to 70 °C in 10 °C intervals using a Jasco V-650 spectrophotometer. For thin film spectra, polymer films were prepared by spin-coating polymer solutions with a concentration of 3 mg/mL onto quartz substrates. Cyclic voltammetry (CV) was measured by a CHI 627E electrochemical analyzer consisting of a three-electrode cell system, where ITO was used as the working electrode and platinum wire was used as the auxiliary electrode. Ag/AgCl and KCl (saturated) reference electrodes were used to determine the cell potential. The electrochemical properties of polymer films were measured in anhydrous acetonitrile with 0.1 M tetrabutylammonium perchlorate as the electrolyte.

The mechanical properties and stretchability of polymer films were investigated using the transferred and stretched method. Polymer films were first prepared by spin-coating on ODTS-modified SiO2/Si substrates. The prepared films were then transferred and printed onto an elastomeric poly(dimethylsiloxane) (PDMS) (base/cross-linker = 17:1 w/w) slab. The polymer/PDMS matrix was then subjected to different strains from 0 to 100%, placed on a cleaned glass slide, and the morphological changes within the strain levels were captured using an optical microscope. Note that the polymer films used to fabricate FETs and for UV, CV, AFM, DMT and GIWAXS analysis were all prepared using the same spin-coating method.

## Supporting Information Available

Synthetic routes of monomers and polymers; calculation method of mobility and paracrystallinity; temperature-dependent UV–vis absorption spectra of polymer solutions; FET characteristics, morphological analyses (DMT, OM images, and GIWAXS), and relevant crystallographic parameters of the studied polymers(PDF)
