# Supplementary material for: The Influence of Interlocking Effects in Conjugated Polymers Synthesized by Aldol Polycondensation on Field-Effect Transistor Properties and Morphology
Source: JACS Au. 2025 Feb 27;5(3):1382–91. doi: 10.1021/jacsau.5c00003 (PMC11938031; doi:10.1021/jacsau.5c00003)
Supplement: Supplementary file 1 — au5c00003_si_001.pdf [file au5c00003_si_001.pdf]

Supporting Information

The Influence of Interlocking Effects in Conjugated Polymers Synthesized by Aldol Polycondensation on Field-Effect Transistor Properties and Morphology

Yen-Han Shih,[a] *Guan-Lin Wu,*[a] *Pin-Hsiang Chueh,*[a] *Jing-Chun Chen,*[a] *Chu-Yen Tsai,*[a] *Ting-Yu Wang,*[a] *Ming-Hsuan Yu,*[a] *Yi-Pei Li,*[a] *Wen-Chang Chen,*[a,b] *Chu-Chen Chueh*[a*]

[a] Y.-H. Shih, G.-L. Wu, P.-H. Chueh, J.-C. Chen, C.-Y. Tsai, T.-W. Wang, M.-H. Yu, Prof. Y.-P. Li, Prof. W.-C. Chen, and Prof. C.-C. Chueh
Department of Chemical Engineering, National Taiwan University, Taipei 10617, Taiwan

[b] Prof. W.-C. Chen
Advanced Research Center for Green Materials Science and Technology, National Taiwan University, Taipei 10617, Taiwan

[*]Corresponding author. E-mail: cchueh@ntu.edu.tw

Keywords: Organic field-effect transistor; aldol condensation; ladder-type conjugated polymers; ambipolar; DFT calculations

■ **Synthesis of monomers**

***Synthesis of 6-bromo-1-(2-octyldodecyl)indoline-2,3-dione* (1).** 6-bromoindoline-2,3-dione (2.0 g, 8.85 mmol) and potassium carbonate (1.712g, 12.39 mmol) were added into a double-neck flask and dissolved in dehydrated dimethylformamide (DMF) (50 mL) under nitrogen protection. 9-(iodomethyl)nonadecane (6.426 g, 15.92 mmol) was injected into the double-neck flask via a syringe. The flask was heated to 100˚C and stirred for 18 hours. The mixture was extracted with ethyl acetate (EA) and brine, and the organic phase was separated, dried with magnesium sulfate, and dark brown oil crude products were obtained. The crude products were then purified by column chromatography using ethyl acetate:hexane (1:5) as the eluent. The final products were orange oil (2.816 g, 63 %). ^{1}H NMR (500 MHz, $CDCl_3$) δ 7.43 (d, 1H), 7.24, (d, 1H), 7.01, (d, 1H), 3.55 (d, 2H), 1.23 (br, 45H), 0.86 (tr, 8H).

***Synthesis of 1-(2-octyldodecyl)-6-(4,4,5,5-tetramethyl-1,3,2-dioxaborolan-2-yl)indoline-2,3-dione* (2).** 6-bromo-1-(2-octyldodecyl)indoline-2,3-dione (**1**) (2.0 g, 3.95 mmol), bis(pinacolato)diboron (1.2 g, 4.73 mmol), potassium acetate (1.163g, 11.85 mmol), and [1,1′-bis(diphenylphosphino)ferrocene]dichloropalladium (II) (73.7 mg) were added into a microwave vial and sealed under nitrogen protection. 1,4-dioxane (1.3 mL) was injected into the vial using a syringe. The vial was then heated

in a microwave reactor at 120 °C for 1 hr. The mixture was extracted with EA and brine, and the organic phase was separated and dried over magnesium sulfate. The crude products were then purified by column chromatography using dichloromethane:ethyl acetate (4:1) as the eluent. The final products were orange solids (865 mg, 39.6 %). ^{1}H NMR (500 MHz, $CDCl_3$) δ 7.54 (d, 2H), 7.22 (d, 1H), 3.59 (d, 2H), 1.34-1.23 (br, 51H), 0.85 (tr, 8H).

Synthesis of 1-(2-octyldodecyl)-6-(4,4,5,5-tetramethyl-1,3,2-dioxaborolan-2-yl)indoline-2,3-dione **(M1). 1** (461 mg, 0.91 mmol), 1-(2-octyldodecyl)-6-(4,4,5,5-tetramethyl-1,3,2-dioxaborolan-2-yl)indoline-2,3-dione (**2**) (550 mg, 0.99 mmol), and [1,1′-bis(diphenylphosphino)ferrocene]dichloropalladium (II) (10 mg) were sealed in a microwave vial under nitrogen protection. Potassium carbonate (55 mg, 0.4 mmol) was dissolved in a small amount of water and injected into the vial via a syringe. Acetonitrile (4 mL) was also injected into the vial via a syringe, and the vial was heated to 80°C in a microwave reactor for 60 mins. The mixture was extracted twice with EA and brine, and the organic phase was collected and dehydrated with magnesium sulfate. The crude products were purified by column chromatography using ethyl acetate:hexane (1:3) as the eluent. The target products were reddish orange solids (225, 29%). ^{1}H NMR (500 MHz, $CDCl_3$) δ 7.71 (d, 2H) 7.28 (d, 2H), 6.98 (d, 2H), 3.65 (d, 4H), 1.87 (s, 3H), 1.21

(br, 32), 0.85 (tr, 6H).

***Synthesis of 3,7-dihydrobenzo[1,2-b:4,5-b']difuran-2,6-dione* (M2).** 2,2'-(2,5-dihydroxy-1,4-phenylene)diacetic acid (1.0 g, 4.42 mmol), toluene (50 mL) and acetic anhydride (10 mL) were added in a flask. The mixture was heated at 100°C for 5 hrs. The mixture was dried using a rotary evaporator and purified by column chromatography using pure chloroform as the eluent. The products were grayish white solids (567mg, 67.4%). ^{1}H NMR (500 MHz, $CDCl_3$) δ 7.05 (s, 2H), 3.76 (s, 4H).

■ **General procedure for polymerization**

M1, and/or 4,4,9,9-tetrahexadecyl-4,9-dihydro-s-indaceno[1,2-b:5,6-b']dithiophene-2,7-dicarbaldehyde (IDT) (**M3**) (1.0 eq molar), and/or **M2** (1.0 eq molar), and/or **M4** (1.0 eq molar), and 4-methylbenzenesulfonic acid (0.3 eq molar) were dissolved in toluene and sealed in a microwave vial. The vial was bubbled with nitrogen for 15 minutes. Then, depending on the reactants, the experiment was carried out in a microwave reactor at 140°C for several hrs. The details of the experiment are described below. After cooling, the mixture was poured into anhydrous methanol and the precipitates were collected. Then, a Soxhlet extraction was carried out, and the impurities and oligomers were washed with acetone and then with chloroform. Finally,

the final products were collected by precipitated in methanol.

Synthesis of P1. **M1** (85.3 mg, 0.1 mmol), **M2** (19.0 mg, 0.1 mmol), 4-methylbenzenesulfonic acid (5.7 mg, 0.03 mmol), toluene (2 mL). Reacted at 140°C for 8 hrs. Dark blue solids (yield: 87 mg, 88.9%). Anal. Calc'd. for [$C_{67}H_{90}N_2O_6$]: C, 78.94; H, 8.90; N, 2.75; O, 9.42. Found: C, 76.76; H, 9.774; N, 1.77. Molecular weight evaluated by SEC with THF: M_n = 22.5 kDa, M_w = 49.9 kDa, $Đ$ = 2.22.

Synthesis of P2. **M1** (64.1 mg, 0.75 mmol), **M2** (19.0 mg, 0.1 mmol), **M3** (30.5 mg, 0.25 mmol), 4-methylbenzenesulfonic acid (5.7 mg, 0.03 mmol), toluene (2 mL). Reacted at 140°C for 14 hrs. Dark purple solids (yield: 86 mg, 79.5%). Anal. Calc'd. for [$C_{73.5}H1_{02.5}N_{1.5}O_{5.5}S_{0.5}$]: C, 79.45; H, 9.3; N, 1.89; O, 7.92; S, 1.44. Found: C, 78.175; H, 9,181; N, 1.72; S, 1.33. Molecular weight evaluated by SEC with THF: M_n = 22.4 kDa, M_w = 59.4 kDa, $Đ$ = 2.65.

Synthesis of P3. **M1** (42.7 mg, 0.5 mmol), **M2** (19.0 mg, 0.1 mmol), **M3** (61.0 mg, 0.5 mmol), 4-methylbenzenesulfonic acid (5.7 mg, 0.03 mmol), toluene (2 mL). Reacted at 140°C for 14 hrs. Dark purple solids (yield: 91 mg, 81.9%). Anal. Calc'd. for [$C_{80}H_{115}N_1O_5S_1$]: C, 79.88; H, 9.64; N, 1.16; O, 6.65; S, 2.67. Found: C, 78.73; H,

9.617; N, 1.11; S, 2.03. Molecular weight evaluated by SEC with THF: M_n = 18.7 kDa, M_w = 39.0 kDa, $Đ$ = 2.08.

Synthesis of P4. **M1** (21.4 mg, 0.25 mmol), **M2** (19.0 mg, 0.1 mmol), **M3** (91.5 mg, 0.75 mmol), 4-methylbenzenesulfonic acid (5.7 mg, 0.03 mmol), toluene (2 mL). Reacted at 140°C for 14 hrs. Dark purple solids (yield: 88 mg, 74.3%). Anal. Calc'd. for [$C_{86.5}H_{127.5}N_{0.5}O_{4.5}S_{1.5}$]: C, 80.26; H, 9.93; N, 0.54; O, 5.56; S, 3.71. Found: C, 79.47; H, 10.12; N, 0.46; S, 3.25. Molecular weight evaluated by SEC with THF: M_n = 12.1 kDa, M_w = 25.5 kDa, $Đ$ = 2.11.

Synthesis of P5. **M2** (19.0 mg, 0.1 mmol), **M3** (122.0 mg, 0.1 mmol), 4-methylbenzenesulfonic acid (5.7 mg, 0.03 mmol), toluene (2 mL). Reacted at 140°C for 16 hrs. Dark purple solids (yield: 85 mg, 61.1%). Anal. Calc'd. for [$C_{93}H_{140}O_4S_2$]: C, 80.58; H, 10.18; S, 4.63. Found: C, 80.69; H, 9.950; S, 4.14. Molecular weight evaluated by SEC with THF: M_n = 11.4 kDa, M_w = 22.5 kDa, $Đ$ = 1.96.

Synthesis of P6. **M2** (19.0 mg, 0.1 mmol), 6,6,12,12-Tetrakis(4-hexylphenyl)-6,12-dihydrodithieno[2,3-d:2',3'-d']-s-indaceno[1,2-b:5,6-b']dithiophene-2,8-dicarboxaldehyde (**M4)** (107.5 mg, 0.1 mmol), 4-methylbenzenesulfonic acid (5.7 mg,

0.03 mmol), toluene (2 mL). Reacted at 140°C for 14 hrs. Dark purple solids (yield: 85 mg, 61.1%). Anal. Calc'd. for [$C_{84}H_{86}O_4S_4$]: C, 78.34; H, 6.73; O, 4.97; S, 9.96. Found: C, 75.25; H, 6.42; S, 10.07. Molecular weight evaluated by SEC with THF: M_n = 12.8 kDa, M_w = 24.3 kDa, $Đ$ = 1.90.

■ Density functional theory calculation

Molecular geometry. The optimized configuration of the synthesized polymers was determined by Gaussian09W calculations using density functional theory (DFT). The B3LYP method was used with 6-311G(*d*, *p*) basic set for the ground-state molecular simulation of the oligomer consisting of three repeating units, in which the alkyl side chains were trimmed to methyl groups to simplify the simulation. The isovalue for visualization was set to 0.02.

Reorganization energy. The commensurate energies of the synthesized polymers were calculated using DFT in the Q-chem software. The B3LYP method was used with the 6-311G(*d*, *p*) basic set, to model the molecular ground state and single-charged states. To ensure favorable interactions, oligomers consisting of two repeating units were used in the calculations, with the alkyl chains trimmed to methyl groups. The reorganization energy (λ) of hole (λ_{hole}) and electron ($\lambda_{electron}$) were calculated

respectively according to the following equations:

$$\lambda_{electron} = \lambda_{-} + \lambda_{0} = (N_{-} - A_{geo}) + (A_{0} - N_{geo})$$

$$\lambda_{\mathrm{hole}} = \lambda_{+} + \lambda_{0} = (N_{+} - C_{geo}) + (C_{0} - N_{geo})$$

where λ_{-} and λ_{+} are the geometric relaxation energies from the geometry of the neutral state to the geometry of the single charged radical state. λ_0 is the geometrical relaxation energy from the geometry of the single charged radical state to the geometry of the neutral state. A_{geo} and C_{geo} indicate the total energy of the optimized anion/cation geometries. N_{geo} represents the total energy of the optimized neutral geometry. A_0 and C_0 are the total energies of the anion/cation molecule in the optimized neutral geometry, while N_{-} and N_{+} are the total energies of the neutral molecule in the optimized anion/cation radical geometry. The derivation of the reorganization energy can be found in the literature.[1-4]

■ Morphological Characterization

The surface roughness and morphology of small molecule thin films were characterized at room temperature in tapping mode using a 3D Controller atomic force microscope (AFM, Digital Instrument). Surface elastic moduli were assessed using an AFM instrument (Dimension Icon, Bruker) in the PeakForce tapping mode. The surface elastic moduli were assessed for polymer films prepared using the same process for the

FET devices. Cantilevers with spring constants between 5 and 42 N m^{-1}, all equipped with a standard silicon tip (OTESPA), were used. To ensure accurate results in the unloading region (2 to 10 nm), the force set point was adjusted individually for each measurement. Force between 5 and 300 nN were applied during the experiments. The probe oscillation frequency was set to 2 kHz, which is significantly lower than the resonance frequency of the cantilever (300 kHz). The Derjaguin-Muller-Toporov (DMT) model was then used to fit the elastic modulus of polymers under study.

Grazing-incidence wide-angle X-ray scattering (GIWAXS) measurements were performed at the 13A1 beamline of the National Synchrotron Radiation Researcher Center (NSRRC) in Taiwan. X-rays with wavelengths of 1.0271/1.0275Å and an incident angle of 0.12° were used to investigate the crystal structure. The crystallographic coherence length (CCL) is calculated as follows. The CCL is a parameter for analyzing the size of the microcrystals,

$$CCL = \frac{2K}{\Delta_{\mathrm{q}}}$$

where CCL is the coherence length, K is the dimensionless shape factor, which we define as 0.9, and Δ_q is the full width at half maximum of the diffraction peak.

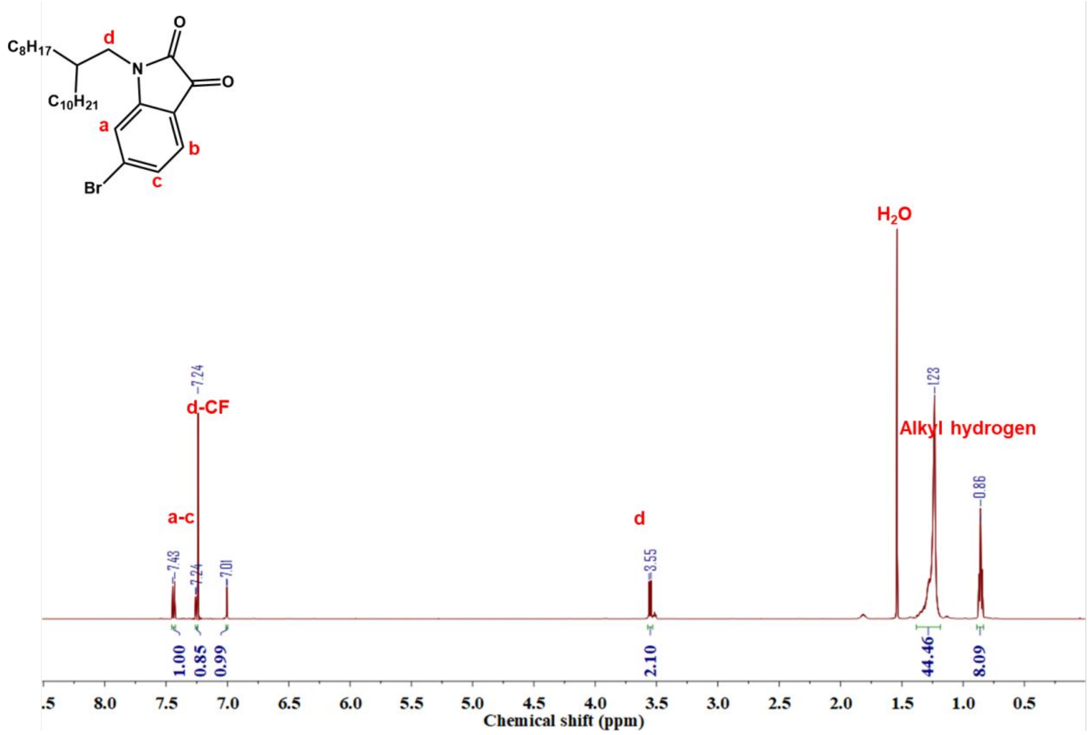

Figure S1. ^{1}H NMR of **1** in $CDCl_3$.

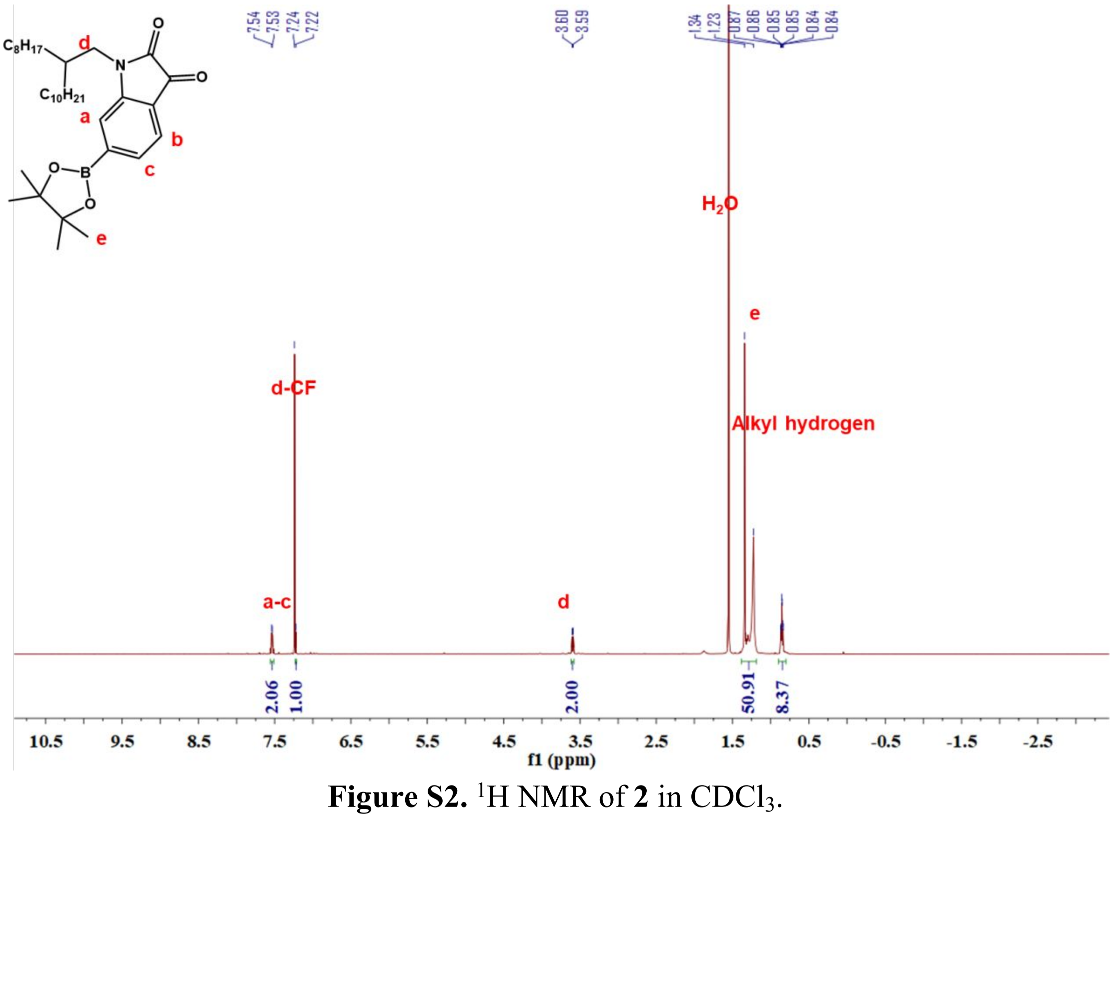

Figure S2. ^{1}H NMR of **2** in $CDCl_3$.

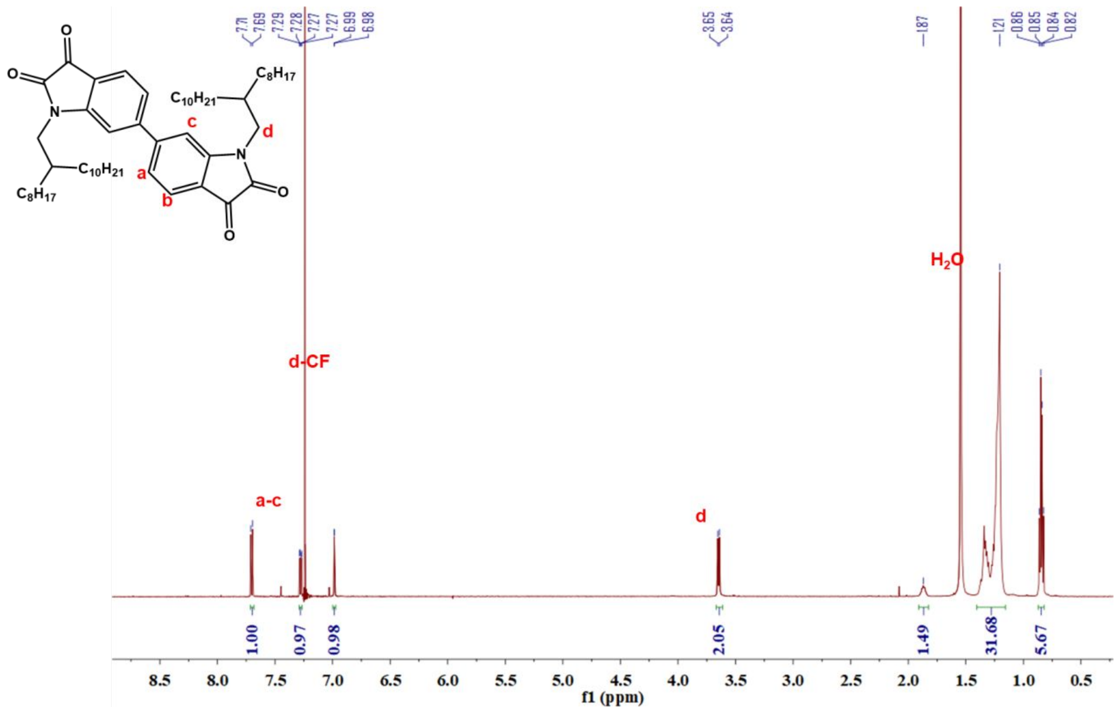

Figure S3. ^{1}H NMR of **M1** in $CDCl_3$.

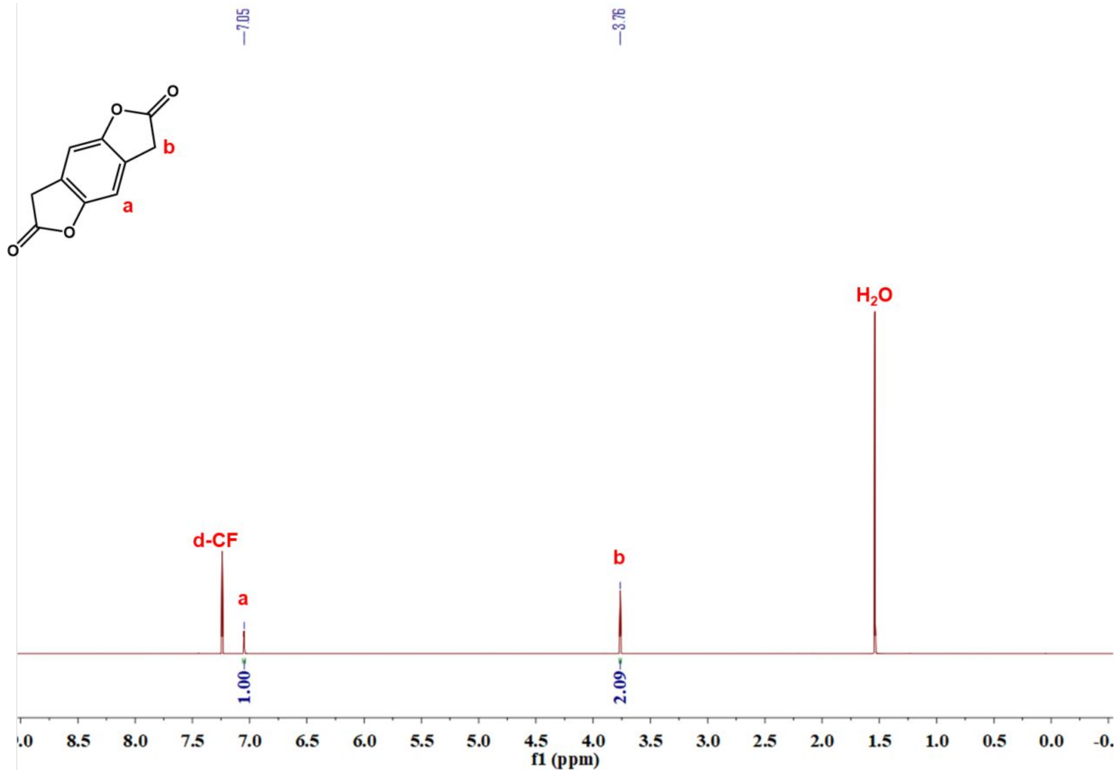

Figure S4. ^{1}H NMR of **M2** in $CDCl_3$.

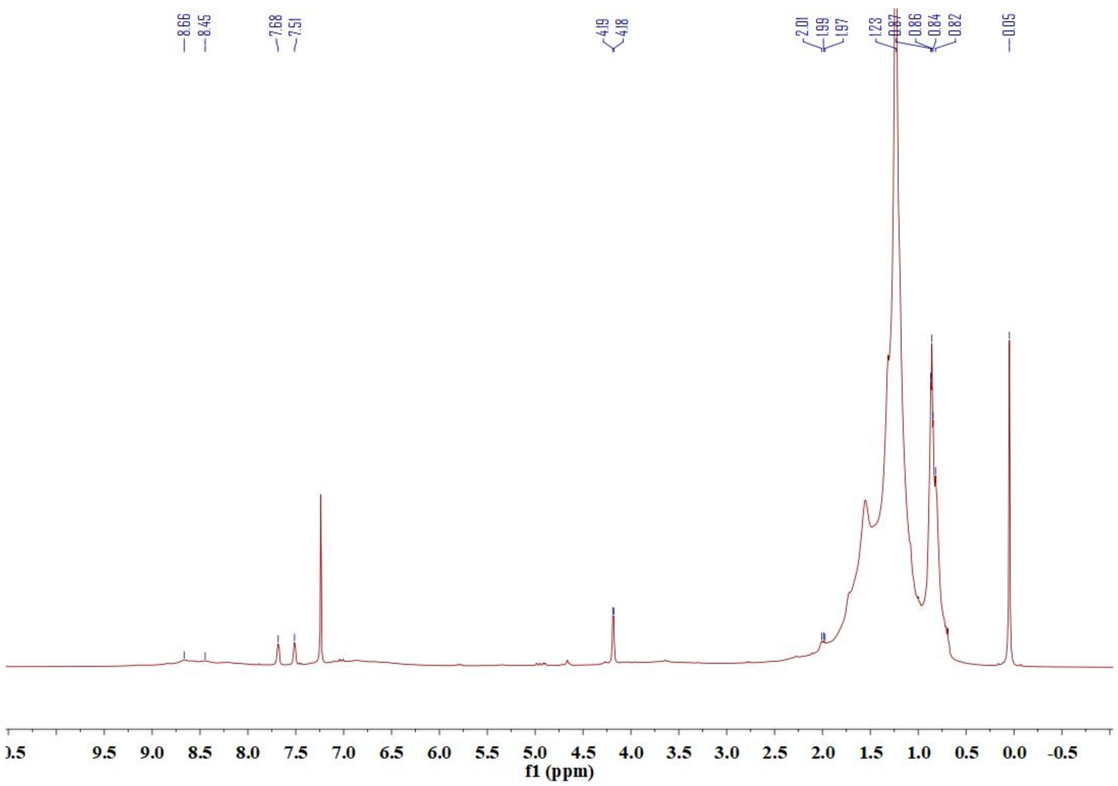

Figure S5. ^{1}H NMR of **P1** in $CDCl_3$.

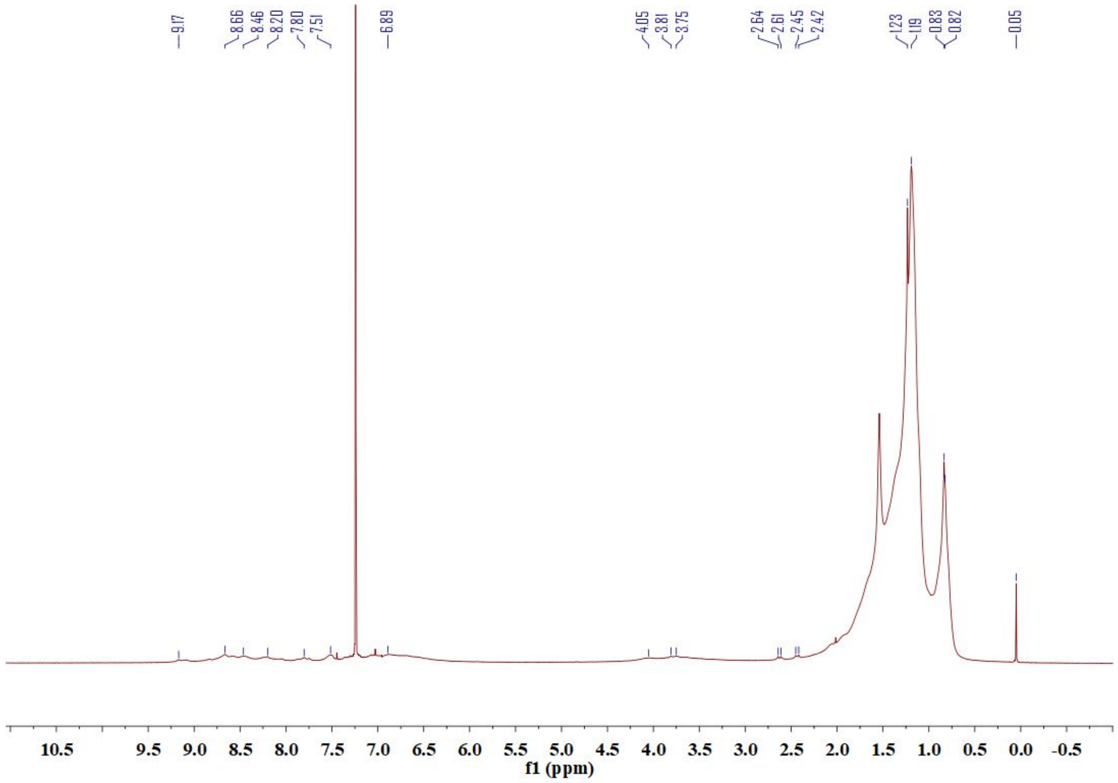

Figure S6. ^{1}H NMR of **P2** in $CDCl_3$.

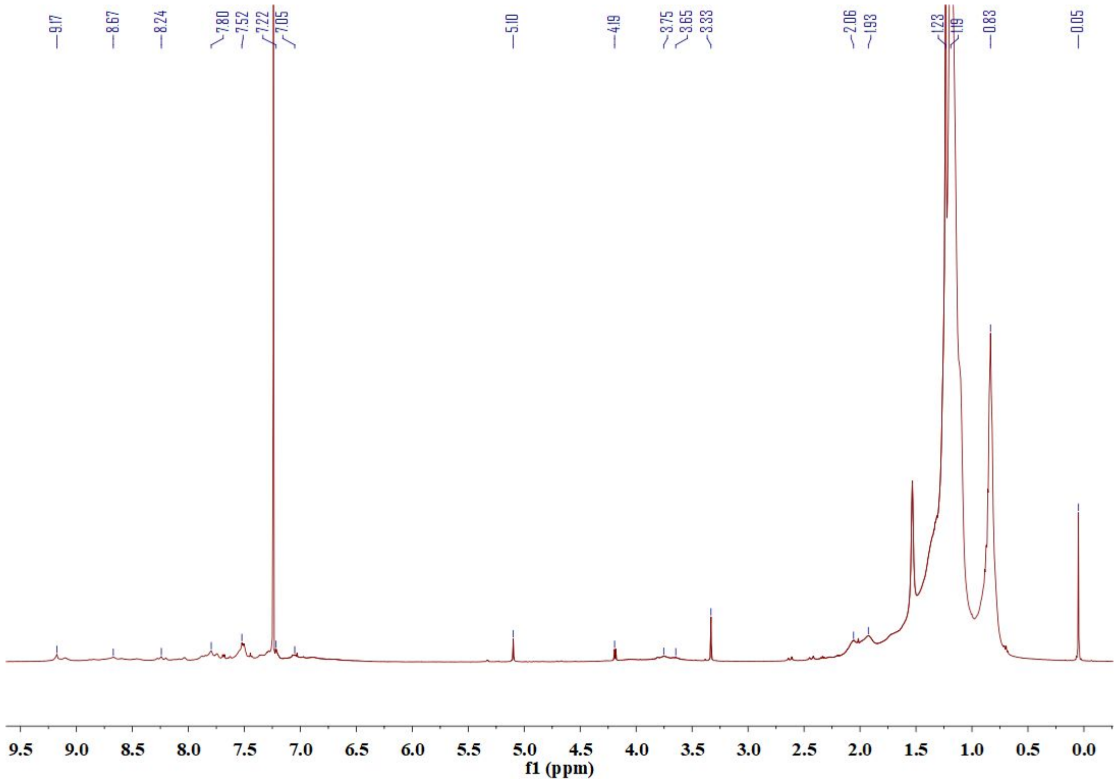

Figure S7. ^{1}H NMR of **P3** in $CDCl_3$.

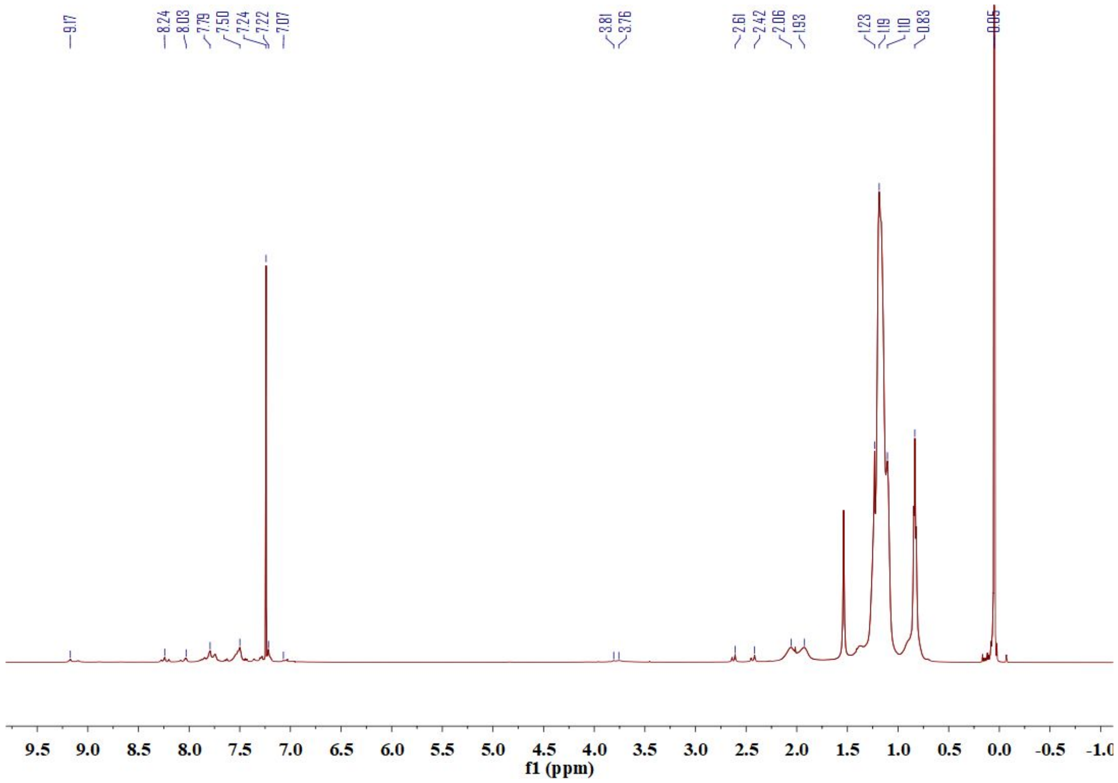

Figure S8. ^{1}H NMR of **P4** in $CDCl_3$.

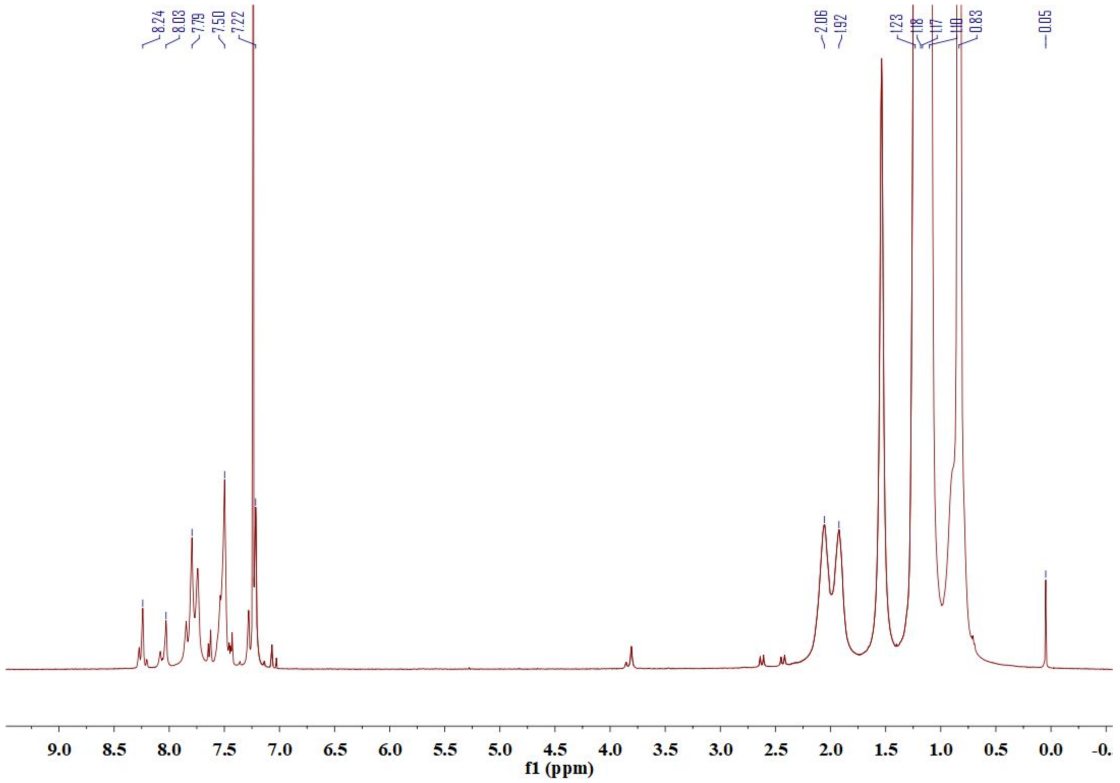

Figure S9. ^{1}H NMR of **P5** in $CDCl_3$.

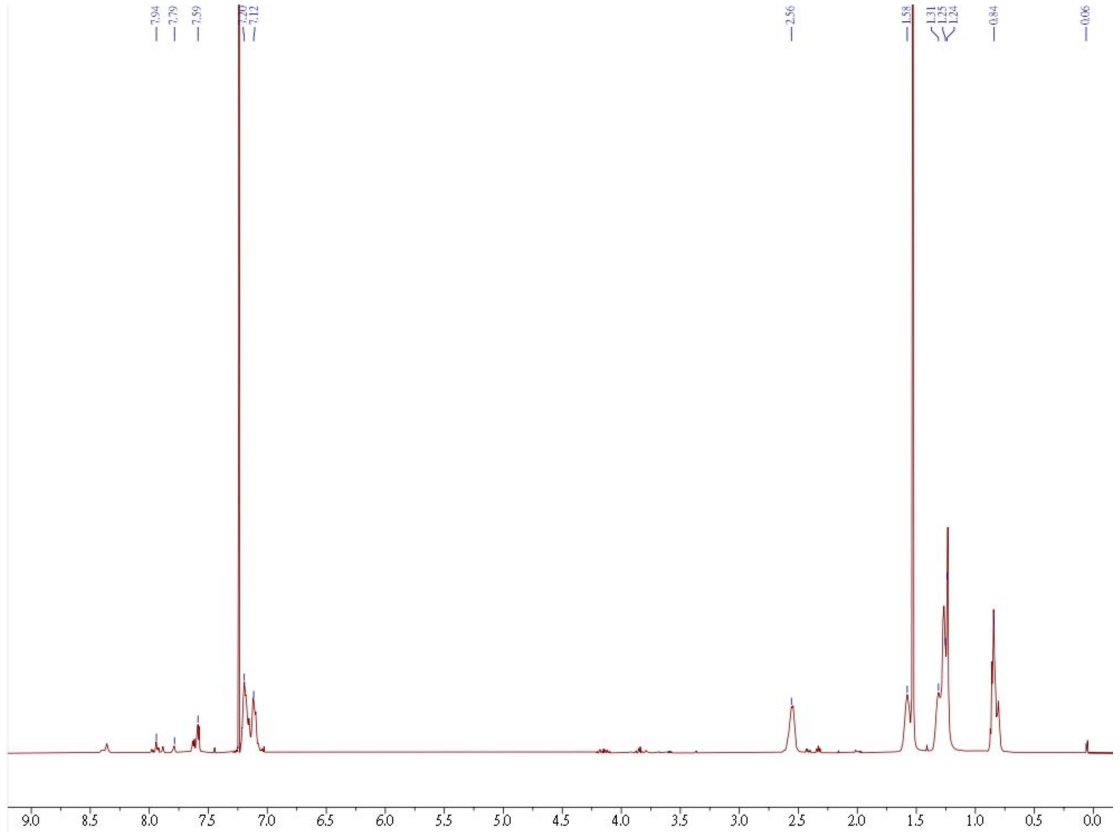

Figure S10. ^{1}H NMR of **P6** in $CDCl_3$.

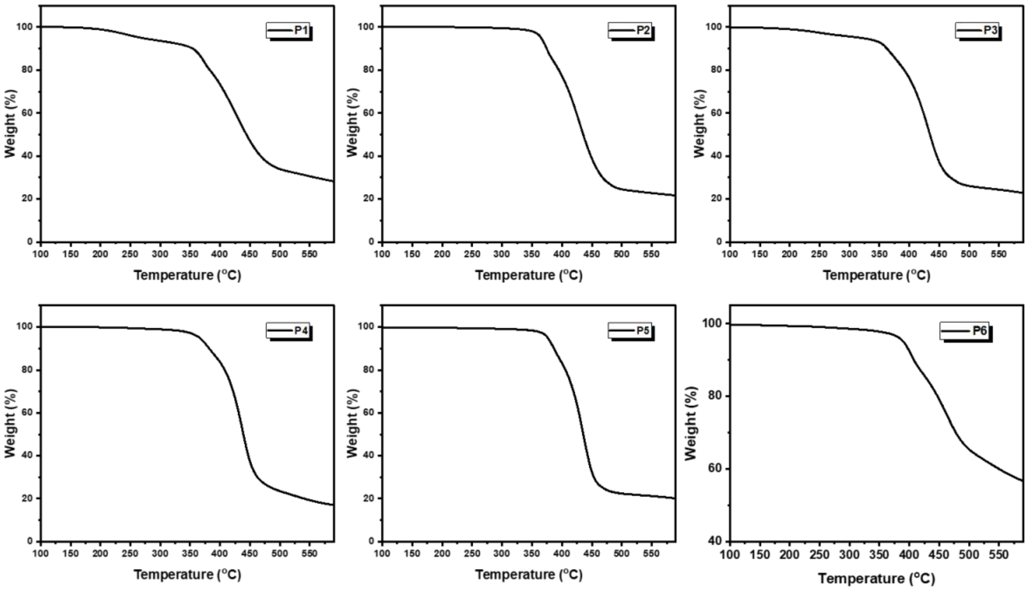

	P1	P2	P3	P4	P5	P6
$T_{d,}5\%$ (°C)	268	365	318	366	376	390

Figure S11. TGA curves of **P1-P6**.

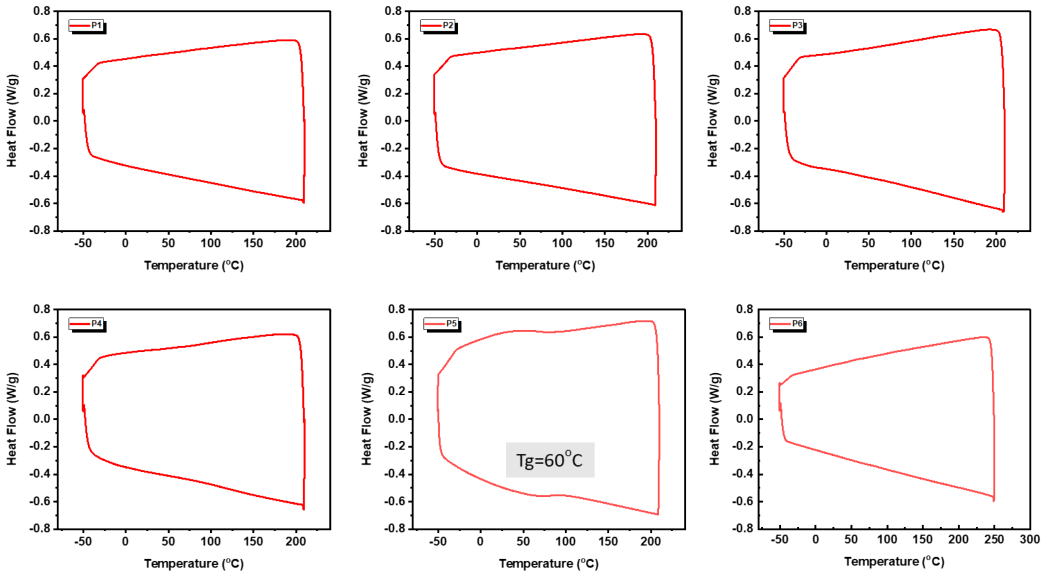

Figure S12. DSC curves of **P1-P6**. The ramping rate of the heating/cooling curves are 10 °C/min.

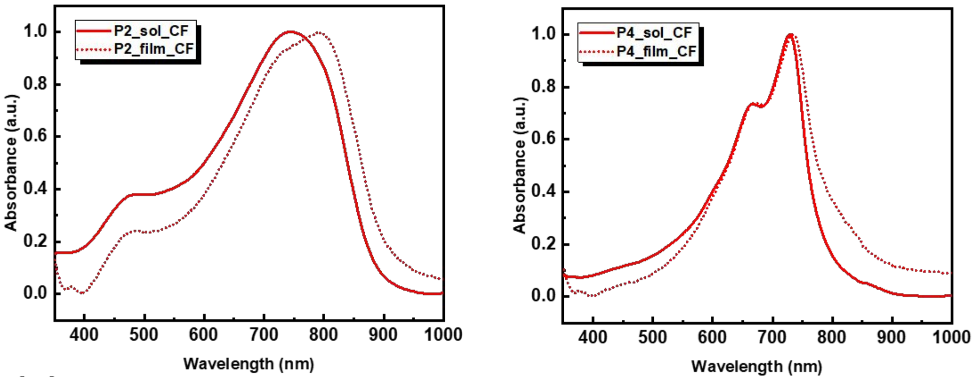

Figure S13. UV-Vis absorption spectra of **P2** and **P4**.

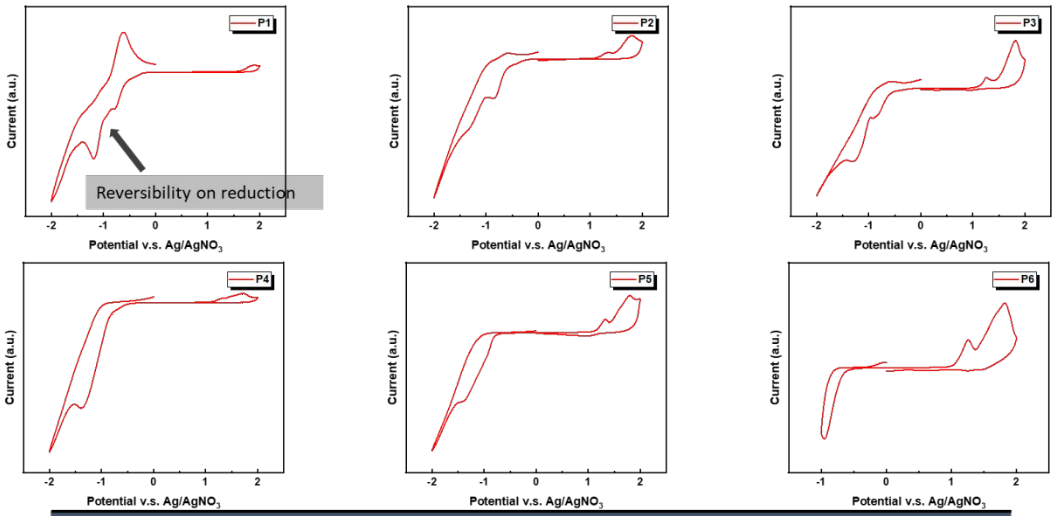

	P1	P2	P3	P4	P5	P6
HOMO(eV)	6.02	5.53	5.53	5.54	5.55	5.45
LUMO(eV)	3.95	3.76	3.79	3.56	3.61	3.72
$E_{g,CV}$(eV)	2.07	1.77	1.74	1.98	1.94	1.73

Figure S14. CV curves and the calculated energy levels for **P1-P6**.

Table S1. The energy of the neutral, cationic, and anionic states of **P5-P6** estimated using DFT calculations.

	**Neutral (hartree)**			**Cation (hartree)**		**Anion (hartree)**	
	**−1(N^-)**	**0(N_{geo})**	**+1(N^+)**	**0(C_0)**	**+1(C_{geo})**	**0(A_0)**	**−1(A_{geo})**
**P5**	-7673.260809	-7673.262551	-7673.260085	-7673.049097	-7673.047328	-7673.369119	-7673.367393
**P6**	-13293.12192	-13293.12311	-13293.12173	-13292.91663	-13292.91454	-13293.22551	-13293.22293

Table S2. The reorganization energy of **P5-P6** estimated using DFT calculations.

	**$\lambda_0(h^+)$ (eV)**	**λ_+ (eV)**	**λ_{hole} (eV)**	**$\lambda_0(e^-)$ (eV)**	**λ_- (eV)**	**$\lambda_{electron}$ (eV)**
	**C_0-N_{geo}**	**$(N_+)-C_{geo}$**	**$\lambda_0(h^+)+\lambda_+$**	**A_0-N_{geo}**	**$(N_-)-A_{geo}$**	**$\lambda_0(e^-)+\lambda_-$**
**P5**	0.067118	0.048146	0.115263	0.047413	0.046957	0.09437
**P6**	0.03745	0.056858	0.094309	0.032318	0.070128	0.102446

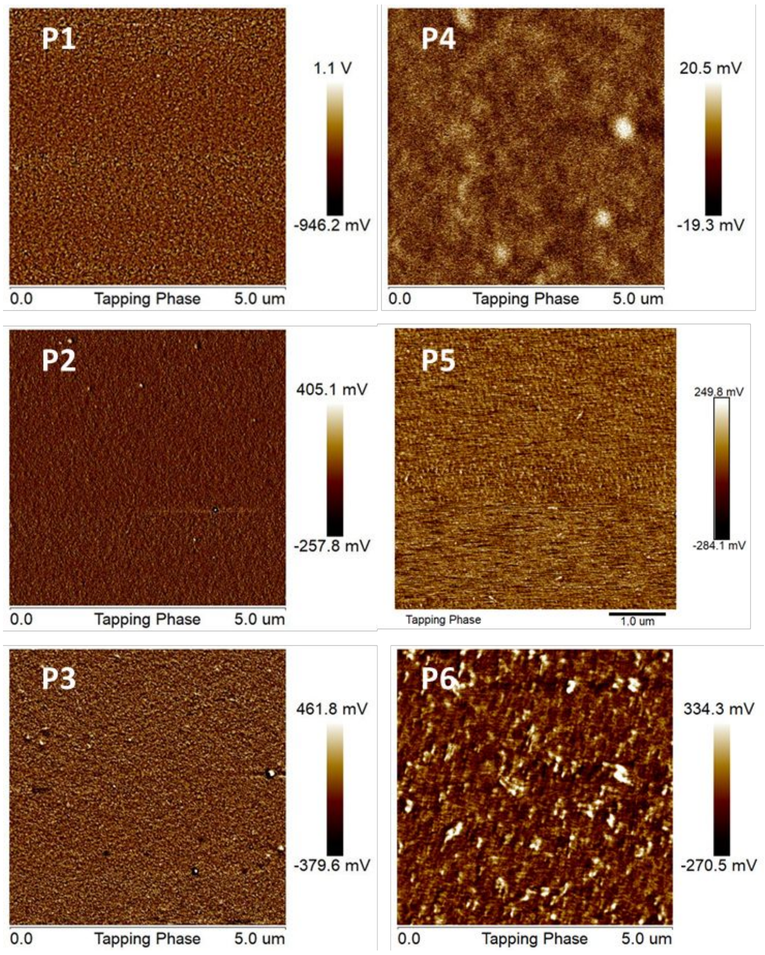

Figure S15. AFM phase images of the **P1-P6** films.

Table S3. Crystallographic properties of the annealed **P1-P6** films.

OOP						
**Material**	**Peak**	q_0	**Stacking distance (Å)**	**FWHM**	**CCL (Å)**	**Paracrystallinity (g%)**
**P1**	**(100)**	0.2930	21.44	0.0461	122.7	15.8
**P2**	**(100)**	0.3051	20.59	0.07985	70.82	20.4
**P3**	**(100)**	0.2933	21.42	0.08109	69.74	21.0
**P4**	**(100)**	0.3172	19.81	0.09914	57.04	22.3
**P5**	**(100)**	0.3974	15.81	0.09022	62.68	19.0
	**(010)**	1.5179	4.14	0.25809	21.91	16.5
**P6**	-	-	-	-	-	-

IP						
**Material**	**Peak**	q_0	**Stacking distance (Å)**	**FWHM**	**CCL (Å)**	**Paracrystallinity (g%)**
**P1**	-	-	-	-	-	-
**P2**	-	-	-	-	-	-
**P3**	-	-	-	-	-	-
**P4**	-	-	-	-	-	-
**P5**	**(010)**	1.4842	4.23	0.19767	28.61	14.6
**P6**	-	-	-	-	-	-

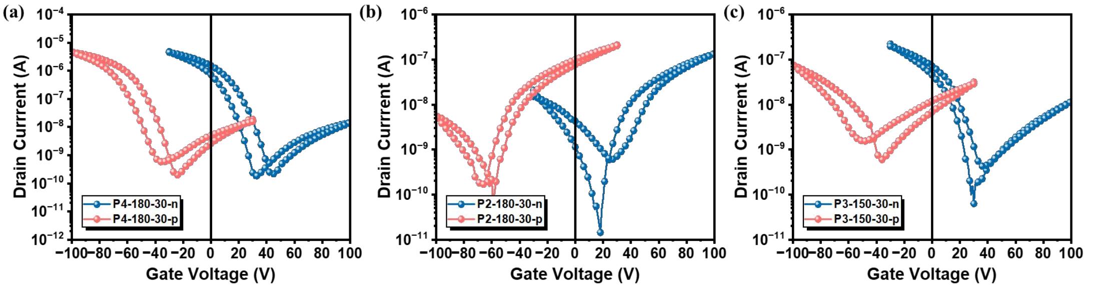

Figure S16. Transfer characteristics of the FET devices based on (a) **P2**, (b) **P3**, and (c) **P4**.

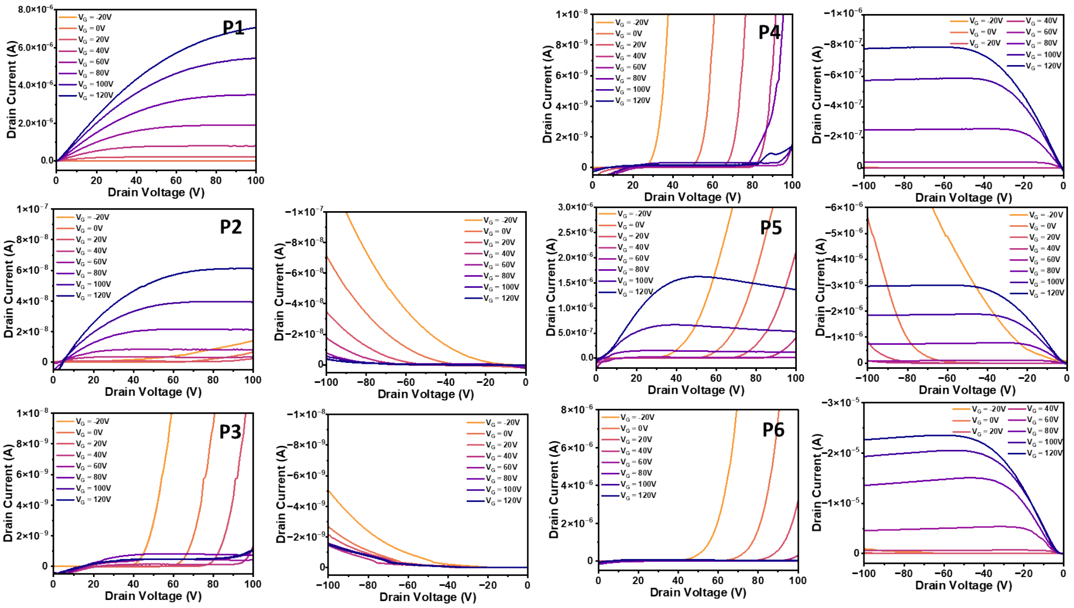

Figure S17. Output curves of **P1-P6** FET devices, showing n-type and p-type characteristics. Note that the p-type characteristic for **P2**, p-type/n-type characteristics for **P3**, and n-type characteristics for **P4** and **P6** are not obtainable due to their low mobility.

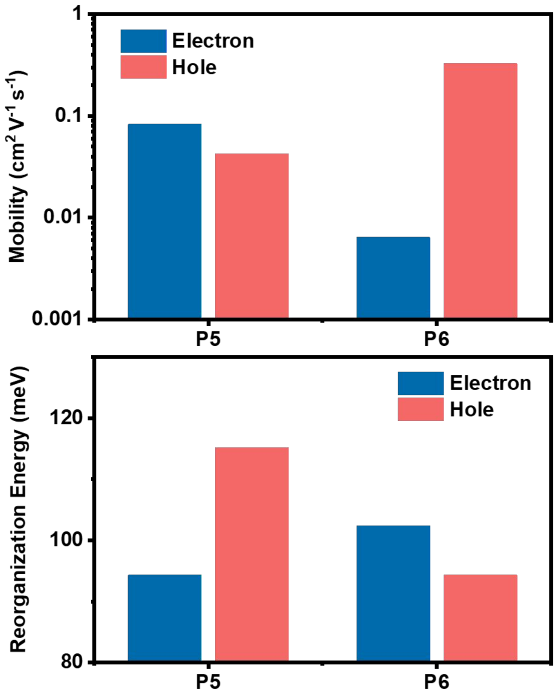

Figure S18. Correlation between the reorganization energy and the mobility performance of **P5** and **P6**.

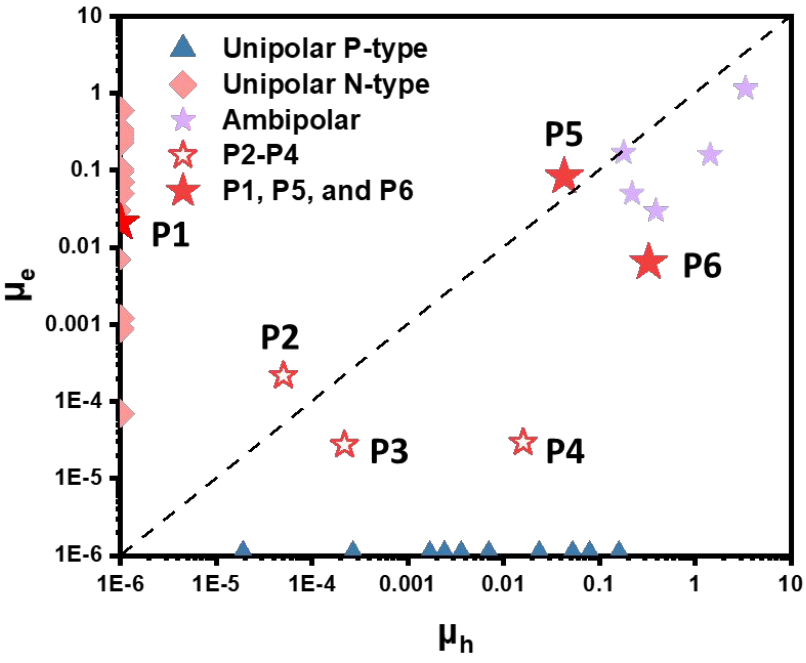

Figure S19. Comparison of charge transport properties of **P1**, **P5**, and **P6** (marked with solid red asterisks) with previous studies of conjugated polymers synthesized using the aldol condensation method.[1, 5-15]

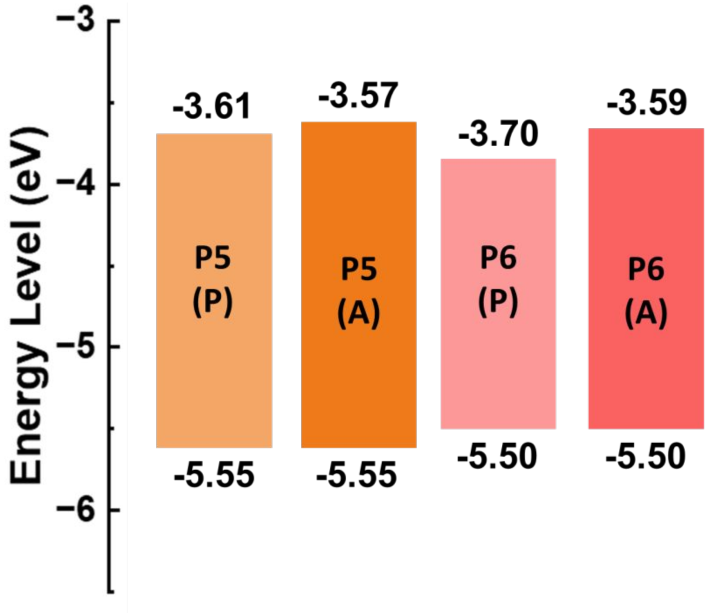

Figure S20. Energy levels of **P5** and **P6** with and without solvent additives. P indicates the pristine state and A indicates the presence of a solvent additive.

Table S4. Crystallographic properties of **P5** and **P6** with and without solvent additives.

	**Condition**	q_0	***d*-spacing (Å)**	**FWHM**	**CCL (Å)**	***g* (%)**
**OOP**	**P5-pristine**	0.39	16.20	0.098	57.51	20.1
		1.47	4.26	0.32	17.49	18.7
	**P5-3%CN**	0.40	**15.52**	0.10	56.20	19.9
		1.51	**4.16**	0.26	21.56	16.6
**IP**	**Condition**	q_0	**π-π stacking distance (Å)**	**FWHM**	**CCL (Å)**	***g* (%)**
	**P5-pristine**	1.40	4.48	0.40	13.96	21.4
	**P5-3%CN**	1.42	4.44	0.39	14.41	20.1

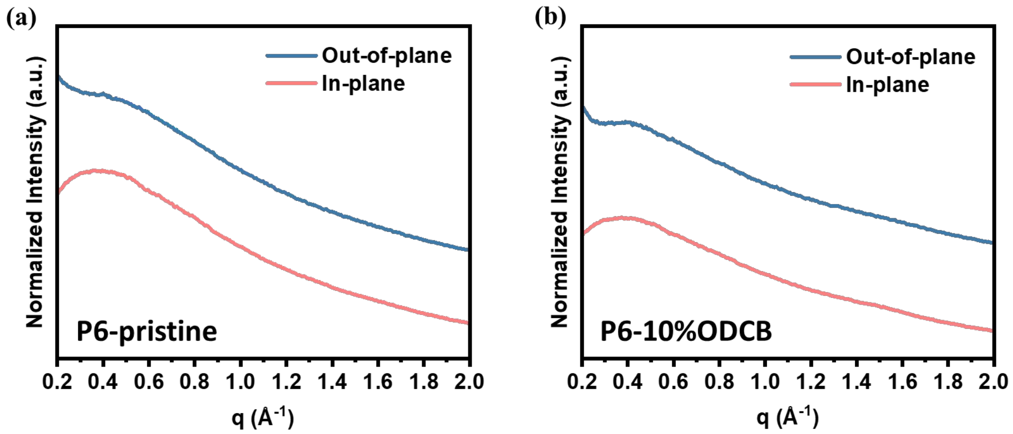

Figure S21. Extracted 1D X-ray scanning curves of **P6** (a) in the pristine state and (b) after the addition of a solvent additive. The absence of a peak indicates the absence of a long-range ordered structure.

Reference

(1) Onwubiko, A.; Yue, W.; Jellett, C.; Xiao, M.; Chen, H.-Y.; Ravva, M. K.; Hanifi, D. A.; Knall, A.-C.; Purushothaman, B.; Nikolka, M.; et al. Fused electron deficient semiconducting polymers for air stable electron transport. *Nat. Commun.* **2018**, *9* (1), 416.

(2) Cortés-Arriagada, D.; Sanhueza, L.; González, I.; Dreyse, P.; Toro-Labbé, A. About the electronic and photophysical properties of iridium(iii)-pyrazino[2,3-f][1,10]-phenanthroline based complexes for use in electroluminescent devices. *Phys. Chem. Chem. Phys.* **2016**, *18* (2), 726-734.

(3) Metri, N.; Sallenave, X.; Plesse, C.; Beouch, L.; Aubert, P.-H.; Goubard, F.; Chevrot, C.; Sini, G. Processable Star-Shaped Molecules with Triphenylamine Core as Hole-Transporting Materials: Experimental and Theoretical Approach. *J. Phys. Chem. C.* **2012**, *116* (5), 3765-3772.

(4) Zade, S. S.; Bendikov, M. Study of Hopping Transport in Long Oligothiophenes and Oligoselenophenes: Dependence of Reorganization Energy on Chain Length. *Chem. Eur. J.* **2008**, *14* (22), 6734-6741.

(5) Zhang, G.; Dai, Y.; Liu, Y.; Liu, J.; Lu, H.; Qiu, L.; Cho, K. Facile green synthesis of isoindigo-based conjugated polymers using aldol polycondensation. *Polym. Chem.* **2017**, *8* (22), 3448-3456.

(6) Alsufyani, M.; Hallani, R. K.; Wang, S.; Xiao, M.; Ji, X.; Paulsen, B. D.; Xu, K.; Bristow, H.; Chen, H.; Chen, X.; et al. The effect of aromatic ring size in electron

deficient semiconducting polymers for n-type organic thermoelectrics. *J. Mater. Chem. C* **2020**, *8* (43), 15150-15157.
(7) Ganguly, A.; He, K.; Hendsbee, A. D.; Abdelsamie, M.; Bennett, R. N.; Li, Y.; Toney, M. F.; Kelly, T. L. Synthesis of Poly(bisisoindigo) Using a Metal-Free Aldol Polymerization for Thin-Film Transistor Applications. *ACS Appl. Mater. Interfaces* **2020**, *12* (12), 14265-14271.
(8) Xiao, M.; Carey, R. L.; Chen, H.; Jiao, X.; Lemaur, V.; Schott, S.; Nikolka, M.; Jellett, C.; Sadhanala, A.; Rogers, S.; et al. Charge transport physics of a unique class of rigid-rod conjugated polymers with fused-ring conjugated units linked by double carbon-carbon bonds. *Sci. Adv. 7* (18), eabe5280.
(9) Guo, Y.; Yang, X.; Wang, L.; Duan, J.; Zhou, Y.; Nielsen, C. B.; Yu, Y.; Yang, J.; Guo, Y.; Li, Z.; et al. Aldol Polymerization to Construct Half-Fused Semiconducting Polymers. *Macromolecules* **2021**, *54* (22), 10312-10320.
(10) Wang, Y.; Zeglio, E.; Wang, L.; Cong, S.; Zhu, G.; Liao, H.; Duan, J.; Zhou, Y.; Li, Z.; Mawad, D.; et al. Green Synthesis of Lactone-Based Conjugated Polymers for n-Type Organic Electrochemical Transistors. *Adv. Funct. Mater.* **2022**, *32* (16), 2111439.
(11) Chen, H.; Moser, M.; Wang, S.; Jellett, C.; Thorley, K.; Harrison, G. T.; Jiao, X.; Xiao, M.; Purushothaman, B.; Alsufyani, M.; et al. Acene Ring Size Optimization in Fused Lactam Polymers Enabling High n-Type Organic Thermoelectric Performance. *J. Am. Chem. Soc.* **2021**, *143* (1), 260-268.
(12) Huang, Y.-W.; Lin, Y.-C.; Li, J.-S.; Chen, W.-C.; Chueh, C.-C. Investigating the backbone conformation and configuration effects for donor–acceptor conjugated polymers with ladder-type structures synthesized through Aldol polycondensation. *J. Mater. Chem. C* **2021**, *9* (30), 9473-9483.
(13) Yu, Y.; Zhu, D.; Zhu, X.; Ravva, M. K.; Duan, J.; Jiang, L.; Li, Z.; Yue, W. A novel class of rigid-rod perylene diimides and isoindigo semiconducting polymers. *Polym. Chem.* **2022**, *13* (4), 536-544.
(14) Tang, H.; Dou, Y.; Tan, R.; Chen, Z.; Liu, C.; Zhang, K.; Zhang, J.; Huang, F.; Cao, Y. N-type conjugated polyelectrolyte enabled by in situ self-doping during aldol condensation. *Polym. J.* **2023**, *55* (4), 517-527.
(15) Che, Q.; Zhang, W.; Wei, X.; Zhou, Y.; Luo, H.; Wei, J.; Wang, L.; Yu, G. High-Mobility Ambipolar Benzodifurandione-Based Copolymers with Regular Donor–Acceptor Dyads Synthesized via Aldol Polycondensation. *CCS Chemistry* **2023**, *5* (11), 2603-2616.
